# ZWINT down-regulated by miR-495-3p inhibited lung metastasis of breast cancer by blocking p38 MAPK signaling pathway activation

**DOI:** 10.1007/s13577-025-01301-z

**Published:** 2025-10-04

**Authors:** Ming-tao Shao, Wei-wen Li, Yong Li, Ping Chen, Shuai-shuai Yu, Wen-jie Lu, Chun-mei Chen, Yan Dong, Yi-wen Zhang, Qun-chen Zhang

**Affiliations:** 1 Department of Breast Medical Center, Jiangmen Central Hospital, Jiangmen, Guangdong China; 2 Department of General Surgery, The First Affiliated Hospital of Jinan University, Guangzhou, Guangdong China; 3 Department of Pathology, Jiangmen Central Hospital, Jiangmen, Guangdong China; 4 Department of Clinical Laboratory, Jiangmen Central Hospital, Jiangmen, Guangdong China; 5 Department of Medical Research Center, Jiangmen Central Hospital, Jiangmen, Guangdong China

**Keywords:** Breast cancer, Lung metastasis, ZWINT, P38 MAPK pathway, MiR-495-3p

## Abstract

Breast cancer metastasis is the primary cause of patient mortality, yet effective therapeutic targets remain limited. Building on our prior identification of ZWINT as a prognostic marker linked to metastasis, this study defines its critical functional role and regulatory mechanism. Multi-omics analysis revealed a strong association between ZWINT expression and metastatic processes across breast cancer subtypes. Functionally, ZWINT knockdown significantly inhibited breast cancer cell migration and invasion in vitro and dramatically reduced lung metastasis in vivo. Mechanistically, we discovered that miR-495-3p directly targets and suppresses ZWINT expression, and this miR-495-3p/ZWINT axis acts through inhibiting p38 MAPK pathway activation to suppress metastatic progression in vitro. Our findings demonstrate that ZWINT drives breast cancer metastasis and is negatively regulated by miR-495-3p. The newly identified miR-495-3p/ZWINT/p38 MAPK axis may provide a promising therapeutic target for suppressing breast cancer progression.

**Supplementary Information:**

The online version contains supplementary material available at 10.1007/s13577-025-01301-z.

## Background

Breast cancer, the most prevalent heterogeneous malignancy among women globally, continues to pose significant threats to women’s health despite advancements in early diagnosis and multimodal therapies [1, 2]. While treatment innovations have improved outcomes, approximately 50% of patients develop metastatic disease [3], which drastically reduces the 5-year survival rate to 26% [4]. Lung metastases account for 60–70% of breast cancer-related deaths [5], often remaining clinically silent until advanced stages and typically emerging within 5 years post-diagnosis [5, 6]. These sobering statistics underscore the urgent need to unravel novel molecular drivers of metastasis and identify actionable therapeutic targets.

Zeste White 10 interactor (ZWINT), a critical kinetochore component, ensures genome stability by regulating centromere–microtubule interactions [7]. Substantial evidence from multiple studies identifies ZWINT as a therapeutic biomarker and oncogenic driver in diverse malignancies [7–9]. For instance, ZWINT serves as a biomarker in malignant glioma, where its knockdown significantly inhibits glioma cell proliferation, invasion, and in vivo tumor growth [10]. Furthermore, ZWINT promotes pancreatic and cervical carcinogenesis by directly interacting with p53 and suppressing the p53/p21 signaling pathway [11, 12]. Currently, research on ZWINT in breast cancer has predominantly examined its role in proliferation [13], and studies have established a strong association between its elevated expression and poor patient prognosis [14, 15]. However, the specific contribution of ZWINT to breast cancer metastasis remains largely unexplored. Interestingly, our previous study has disclosed that ZWINT is significantly associated with distant metastasis-free survival (DMFS) in breast cancer patients, suggesting that ZWINT may be involved in breast cancer metastasis [16]. However, the specific role and underlying mechanisms of ZWINT in breast cancer metastasis remain largely unknown.

MicroRNAs (miRNAs), small non-coding RNAs regulating gene expression via 3′-UTR interactions, orchestrate diverse pathological processes, including metastasis [17, 18]. Among these, miR-495-3p exhibits context-dependent roles in tumorigenesis, functioning as either an oncogene or tumor suppressor across cancer types [19, 20]. In breast cancer, miR-495-3p demonstrates metastasis-suppressive activity by targeting effectors [21], but its regulatory network remains incompletely mapped. Intriguingly, bioinformatic analyses predict conserved miR-495-3p-binding sites within ZWINT 3′-UTR, suggesting a direct regulatory relationship that may underlie metastatic processes.

This study systematically investigates the miR-495-3p/ZWINT axis as an emerging regulator of metastasis in breast cancer. Through integrated in vitro and in vivo approaches, we define functional role of ZWINT in metastatic dissemination beyond proliferation, and elucidate the direct regulation of ZWINT by miR-495-3p via 3′-UTR binding characterize downstream signaling cascades linking this axis to lung tropism. Our findings establish the miR-495-3p/ZWINT/p38 MAPK signaling axis as a therapeutic target with dual diagnostic and therapeutic potential, offering strategies to intercept metastasis before clinical manifestation.

## Materials and methods

### Cell culture

Human normal mammary epithelial cells (MCF-10A) and breast tumor cell lines (MCF-7 and MDA-MB-231) were obtained from the American Type Culture Collection (ATCC). These cells were cultured in RPMI-1640 medium (Pricellla, Wuhan, China) supplemented with 10% fetal bovine serum (FBS) and 1% penicillin–streptomycin. Cells were maintained at 37 °C in a humidified incubator with 5% CO₂. All cell lines were authenticated and tested to ensure the absence of mycoplasma contamination.

### Cell transfection and treatment

The short hairpin RNAs (shRNAs) targeting ZWINT, miR-148-5p mimics/inhibitor, ZWINT overexpression vectors, and their corresponding negative controls were obtained from Sangon Biotech (Shanghai, China). Breast cancer cell lines were cultured until reaching 60–70% confluence and subsequently transfected with these constructs using Lipofectamine™ 3000 (Invitrogen) following the manufacturer's instructions. Transfection efficiency was assessed 24 h post-transfection through quantitative real-time PCR (RT-qPCR) analysis. To investigate the p38 signaling pathway, selected cell populations were exposed to 2 μg/L Anisomycin (Merck) for 24 h prior to analysis. The specific sequences for shRNAs, miR-148-5p mimics/inhibitor, and their negative controls are provided in Supplementary Table 1.

### Total RNA extraction and RT-qPCR analysis

Total RNA was extracted from the indicated cell lines using Trizol reagent (Invitrogen, USA) following the manufacturer’s protocol. Subsequently, the RNA was reverse transcribed into cDNA using the Reverse Transcriptase M-MLV (RNase H-) kit (Takara, Dalian, China). RT-qPCR for mRNA and miRNA was performed on a real-time fluorescent quantitative PCR system (Roche, LightCycler^®^96) using TB Green Premix Ex Taq (Tli RNaseH Plus) (Takara, Dalian, China). The relative expression levels of mRNAs and miRNAs were calculated using the 2^−ΔΔCt^ method, with GAPDH and U6 as internal controls for mRNA and miRNA normalization, respectively. All RT-qPCR primers (Sangon Biotech, Shanghai, China) are provided in Supplementary Table 2.

### Protein extraction and western blot analysis

Total protein was extracted from the designated cell lines and tissues using precooled RIPA lysis buffer (Beyotime, Shanghai, China) supplemented with protease and phosphatase inhibitors. Protein concentrations were quantified using a Detergent Compatible Bradford Protein Assay Kit (Beyotime, Shanghai, China), followed by denaturation. Equal amounts of protein were separated by sodium dodecyl sulfate–polyacrylamide gel electrophoresis (SDS-PAGE) and transferred onto polyvinylidene fluoride (PVDF) membranes (Servicebio, Wuhan, China) using an electrophoresis apparatus (DYY-6C, Beijing 61). The membranes were blocked with 5% nonfat powdered milk (Beyotime, Shanghai, China) in TBST for 2 h at room temperature on a horizontal shaker. After blocking, the membranes were incubated overnight at 4 °C with the following primary antibodies: anti-ZWINT (Proteintech, 12282-2-AP, 1:1000), anti-Phospho-p38 MAPK (Zenbio, SR43-04, 1:1000), anti-p38 MAPK (HUABIO, ER65580, 1:1000), and anti-β-actin (Proteintech, 20536-1-AP, 1:5000). Subsequently, the membranes were incubated with horseradish peroxidase (HRP)-conjugated secondary antibody (DING GUO, IH-0011, 1:5000) for 2 h at room temperature. Protein bands were visualized using an Enhanced Chemiluminescence (ECL) kit (DING GUO, Beijing, China) on a chemiluminescence imaging system (Bio-Rad, USA) and quantified using ImageJ software.

### Wound-healing assay

Cell migration ability was assessed using a wound-healing assay. Briefly, confluent monolayers of breast cancer cells were scratched with a sterile 10 μL pipette tip to create a uniform wound. Images of the wound area were captured at 0 h and 24 h post-scratch using an inverted microscope (Leica DMI1, Germany). The wound area was quantified using ImageJ software (NIH, USA), and the migration rate (%) was calculated as follows: Migration rate (%) = [(0 h wound area − 24 h wound area)/0 h wound area] × 100.

### Transwell invasion assay

Cell invasion capacity was evaluated using Transwell inserts (Corning, NY, USA) precoated with Matrigel (50 μg/well). Briefly, 1 × 10^5^ cells suspended in serum-free medium were seeded into the upper chamber, while the lower chamber was filled with medium containing 20% FBS as a chemoattractant. After 24 h of incubation, non-invading cells on the upper membrane surface were removed with a cotton swab. Invaded cells on the lower surface were fixed with 4% paraformaldehyde (Beyotime, China) for 15 min, stained with 0.1% crystal violet (Beyotime, China) for 30 min, and imaged under an inverted microscope (Nikon Eclipse Ts2, Japan). Five random fields per insert were counted to quantify invasive cells.

### Luciferase report assay

The dual-luciferase reporter gene assay was employed to validate the regulatory interaction between ZWINT and miR-495-3p. Initially, the wild-type (WT) and mutant (Mut) sequences of ZWINT, the latter containing a mutation at the potential miR-495-3p binding site, were cloned into pmirGLO vectors to generate the respective luciferase reporter constructs (ZWINT-WT and ZWINT-Mut). Subsequently, breast cancer cell lines were co-transfected with either miR-495-3p mimics or negative control (NC) mimics, along with ZWINT-WT or ZWINT-MUT, using Lipofectamine™ 3000 (Invitrogen). Following 24 h of co-transfection, luciferase activity was quantified using the dual-luciferase reporter assay system (Promega, USA) in accordance with the manufacturer's protocol. The relative luciferase activity was calculated as the ratio of Firefly luciferase activity to Renilla luciferase activity.

### Generation of stable ZWINT knockdown cell lines

Stable knockdown of ZWINT was constructed using lentiviral vectors pLKO.1-puro (Hanbio, Shanghai, China). In brief, HEK293T cells were co-transfected with pLKO.1-puro encoding ZWINT-targeting shRNA (shZWINT) or a non-targeting scramble shRNA (shscramble), along with the lentiviral packaging plasmids psPAX2 and pMD2.G, using LV-MAX™ transfection kit (Invitrogen). Viral supernatants were harvested at 48 h post-transfection, filtered, and concentrated by ultracentrifugation (25,000×*g*, 2 h, 4 °C), and viral titer was determined via Lenti-X™ qRT-PCR Titration Kit (Takara, Dalian, China). Subsequently, MDA-MB-231 cells were infected with the corresponding concentrated ZWINT knockdown lentiviruses (LV-shZWINT) and scramble shRNA lentiviruses (LV-shscramble) in the presence of 8 µg/mL polybrene (Sigma-Aldrich). Following a 48-h incubation, infected cells were selected with 10 μg/mL puromycin (MCE, USA) for 3 days to establish stable ZWINT knockdown cells. Knockdown efficiency was verified by RT-qPCR.

### Mouse models of lung metastasis

To establish the breast tumor metastasis model, BALB/C nude mice (4 weeks old, female) were purchased from Guangdong Medical Laboratory Animal Center (Guangzhou, China). After 7 days of cage adaptation under specific pathogen-free (SPF) conditions in the animal experiment center, these mice were randomly divided into LV-shZWINT group (*N* = 6) and LV-shscramble group (*N* = 6). The designated stable cell lines (2 × 10^6^ cells/mL, 100 μL/mouse) were injected into the tail vein of each group to establish the lung metastasis model of nude mice. Eight weeks after tail vein injection, the mice were euthanized with carbon dioxide and then harvested their lungs. After counting the tumor nodules in the lung under a stereoscopic microscope, part of the mice was fixed in 4% paraformaldehyde overnight for hematoxylin and eosin (HE) staining, and the rest of the samples were immediately frozen in liquid nitrogen and stored in a refrigerator at −80℃ for molecular biological analysis. All animal experiments were cared for in accordance with the ethical standards and national guidelines, and approved by the Animal Ethics Committee of Guangdong Medical University (GDY2102365).

### HE staining

Fixed lung tissue samples were dehydrated, permeabilized, embedded in paraffin, and sectioned for HE staining. Briefly, paraffin sections were deparaffinized with xylene, hydrated with gradient ethanol, and stained with hematoxylin staining solution (Beyotime, Shanghai, China). After staining for 10 min, the sections were differentiated with 1% alcohol hydrochloride for 1 min and then returned to blue with 0.6% ammonia. Next, the sections were stained for 5 min in 0.5% eosin staining solution (Beyotime, Shanghai, China). Finally, the sections were dehydrated, transparent and sealed, and then photographed and observed for structural changes in lung tissue under a microscope (Leica, DMT1).

### Bioinformatics analysis

We utilized the CancerSEA online database (http://biocc.hrbmu.edu.cn/CancerSEA/) to evaluate the average correlation between ZWINT and the biological functions of breast cancer cells. To predict miRNAs potentially binding to ZWINT, we employed multiple tools, including miRanda (http://www.miranda.org/), miRmap (https://mirmap.ezlab.org/), miRcoT (http://diana.imis.athena-innovation.gr/DianaTools/index.php?r=mrmicrot/index), and PITA (http://genie.weizmann.ac.il/pubs/mir07/mir07_data.html). The correlation between ZWINT expression levels and miRNAs in breast cancer tissues was assessed using breast cancer data from The Encyclopedia of RNA Interactomes (ENCORI) (http://starbase.sysu.edu.cn/index.php). Kaplan–Meier plotter (https://kmplot.com/analysis/index.php?p=home) was used to assess the correlation between the expression of miR-495-3p and survival in tissue samples from patients with breast cancer.

### Statistical analysis

All data, derived from at least three biological replicates, are presented as the mean ± standard deviation (SD). One-way ANOVA and Student's *t* test were used to compare multiple groups and two groups, respectively. Statistical analysis and graph plotting were performed using GraphPad Prism 6, with statistical significance set at *P* < 0.05.

## Results

### ZWINT knockdown inhibits migration and invasion of breast cancer cells in vitro

**Fig. 1:**
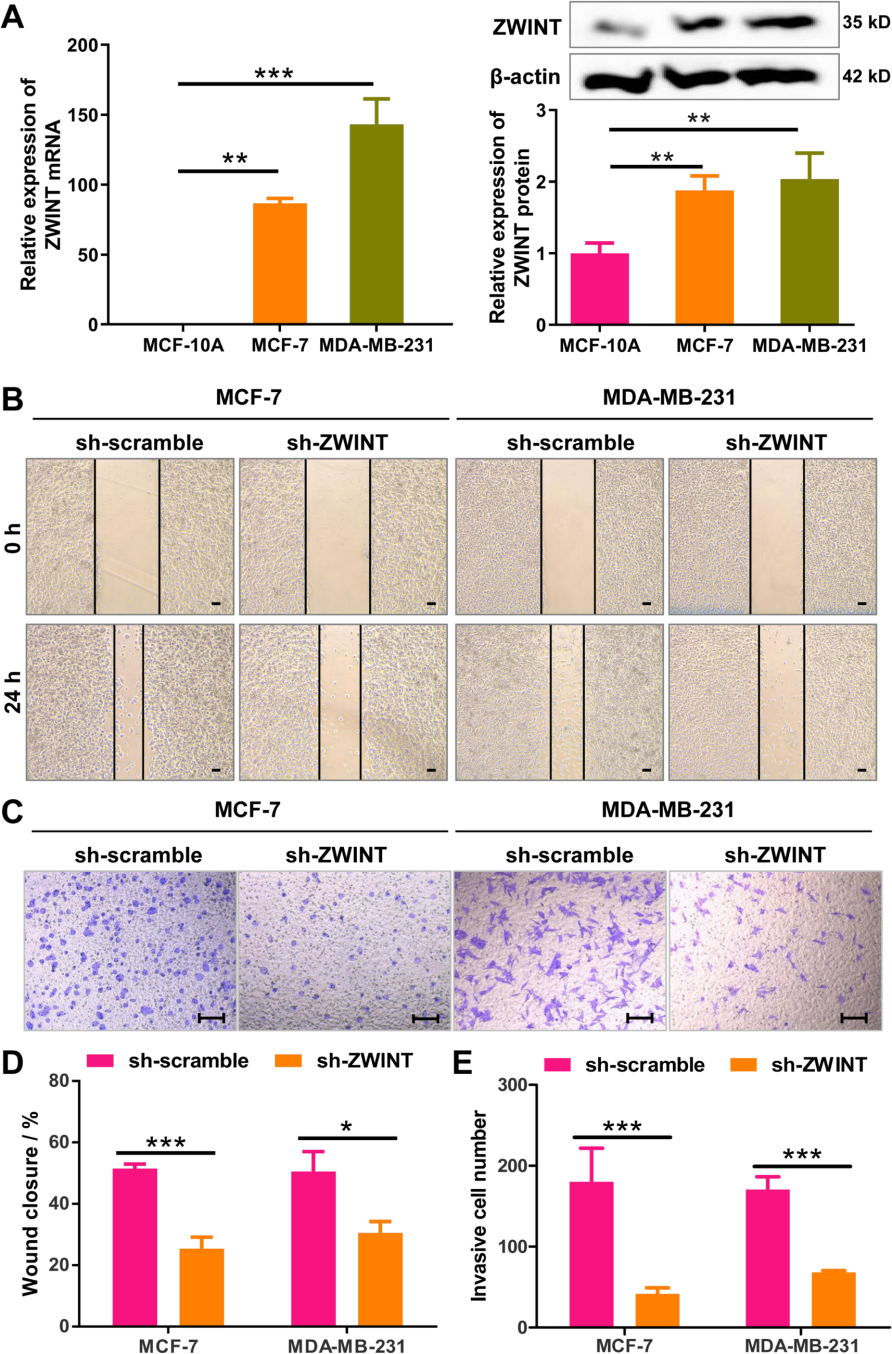
ZWINT knockdown inhibits migration and invasion of breast cancer cells in vitro. **A** RT-PCR and western blot analysis of ZWINT mRNA and protein levels in MCF-10A, MCF-7, and MDA-MB-231 cells. **B**, **D** Wound-healing assay showed the migration rates of the control and ZWINT-silenced MCF-7/MDA-MB-231 cells. Bar = 100 μm. **C**, **E** Transwell invasion assay showing decreased invasion rates in ZWINT-silenced MCF-7 and MDA-MB-231 cells compared to controls. Bar = 100 μm. *N* = 3, *P < 0.05, **P < 0.01, and ***P < 0.001

We first evaluated the average correlation between ZWINT and the biological functions of breast cancer cells using the CancerSEA database. The results showed that ZWINT was closely associated with the invasion of breast cancer cells, in addition to regulating their proliferation (Supplementary Fig. 1). Next, we analyzed ZWINT expression levels in the human normal mammary epithelial cell line (MCF-10A) and human breast tumor cell lines (MCF-7 and MDA-MB-231) using RT-qPCR and western blot. The results indicated that ZWINT expression was significantly higher in MCF-7 and MDA-MB-231 cells at both the mRNA and protein levels (Fig. 1A). To further investigate the role of ZWINT in the migration and invasion of breast cancer cells, we constructed three shRNAs specifically targeting ZWINT (shZWINT-1, shZWINT-2, and shZWINT-3) and assessed their interference effects. RT-qPCR and western blot results showed that all three shRNAs significantly inhibited ZWINT expression in breast cancer cells, with shZWINT-1 exhibiting the most potent inhibitory effect and thus selected for subsequent experiments (Supplementary Fig. 2). Wound-healing assay revealed that ZWINT knockdown significantly inhibited cell migration in MCF-7 and MDA-MB-231 cells (Fig. 1B, D). Similarly, Transwell invasion assay demonstrated that ZWINT knockdown markedly reduced cell invasion in these cell lines (Fig. 1C, E). Collectively, these findings suggest that ZWINT plays a crucial role in promoting the migration and invasion of breast cancer cells.

### ZWINT knockdown suppresses migration and invasion of breast cancer cells by the p38 MAPK signaling pathway

**Fig. 2:**
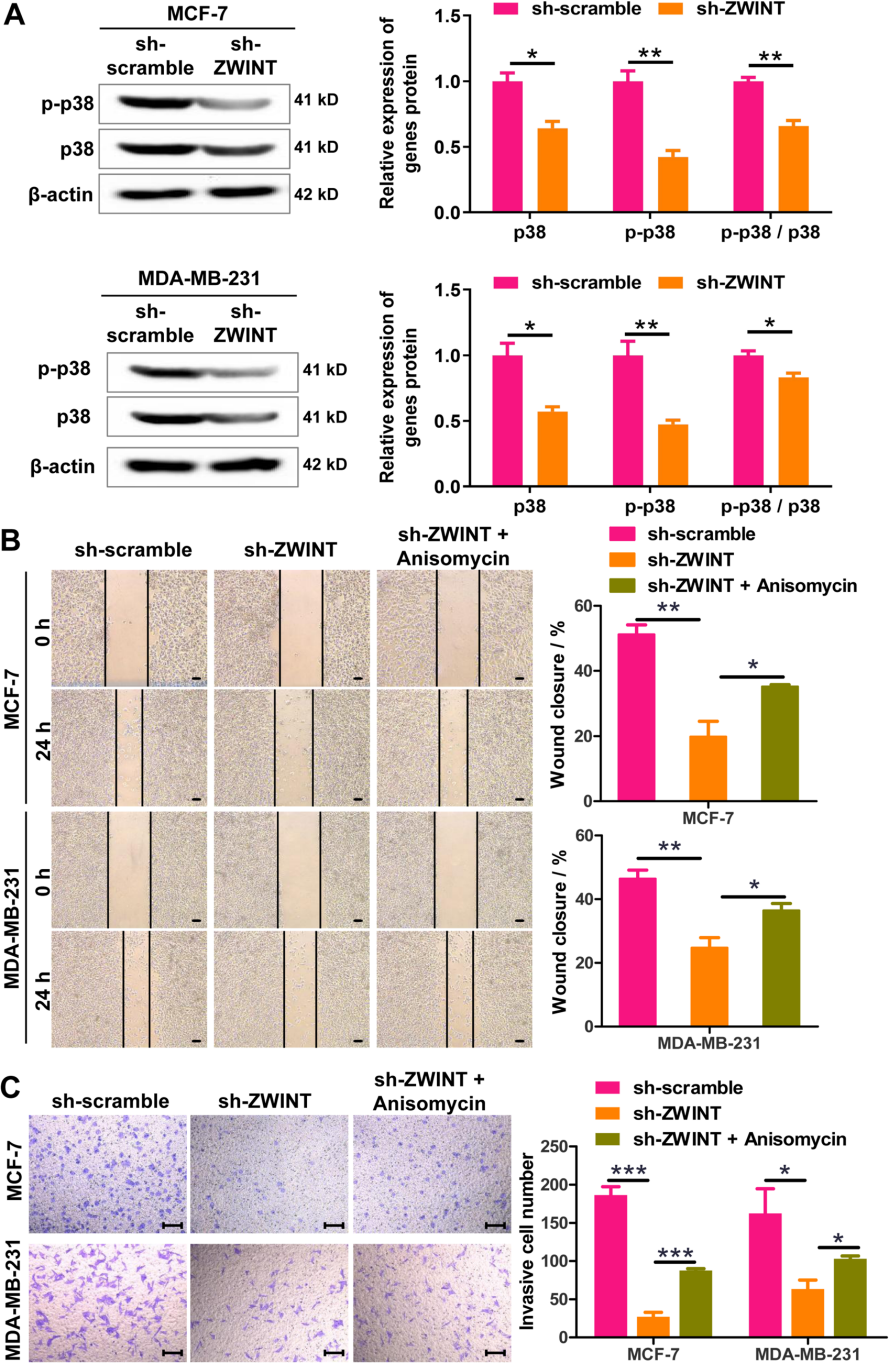
ZWINT knockdown inhibits breast cancer cell migration and invasion by modulating the p38 MAPK pathway. **A** Western blot analysis of p38 MAPK (p38) and phospho-p38 MAPK (p-p38) protein levels in control and ZWINT-silenced MCF-7/MDA-MB-231 cells. **B** Wound-healing assay was used to detect the effect of Anisomycin on the migration of the control and ZWINT-silenced MCF-7/MDA-MB-231 cells. Bar = 100 μm. **C** Transwell chamber invasion assay was used to detect the effect of Anisomycin on the invasion of the control and ZWINT-silenced MCF-7/MDA-MB-231cells. Bar = 100 μm. Anisomycin (2 μg/L), an agonist of p38 MAPK signaling pathway. Bar = 100 μm. *N* = 3, *P < 0.05, **P < 0.01, and ***P < 0.001

Current evidence has shown that the activation of the p38 MAPK signaling pathway plays a crucial role in the metastasis and invasion of breast cancer [22–24]. The results from western blot revealed that ZWINT knockdown significantly inhibited the protein expression levels of p38 MAPK and Phospho-p38 MAPK, as well as significantly reduced the Phospho-p38 MAPK/p38 MAPK ratio in vitro (Fig. 2A), suggesting that ZWINT knockdown inhibits the activation of the p38 MAPK signaling pathway in breast cancer cells. Therefore, we explored whether the p38 MAPK signaling pathway is involved in ZWINT regulation of breast cancer cell metastasis and invasion. We performed rescue experiments with Anisomycin (2 μg/L, the p38 MAPK pathway activator) in MCF-7 and MDA-MB-231 cells. The results from wound healing assay revealed that Anisomycin could significantly weaken the inhibitory effect of ZWINT knockdown on cell migration in MCF-7 and MDA-MB-231 cells (Fig. 2B). Meanwhile, the results from transwell invasion assay indicated that Anisomycin could significantly weaken the inhibitory effect of ZWINT knockdown on invasion of MCF-7 and MDA-MB-231 cells (Fig. 2C). Together, these findings show that ZWINT knockdown inhibits migration and invasion of breast cancer cells by blocking the activation of the p38 MAPK signaling pathway.

### ZWINT knockdown attenuates lung metastasis of breast cancer through inhibiting the activation of p38 MAPK signaling pathway

**Fig. 3:**
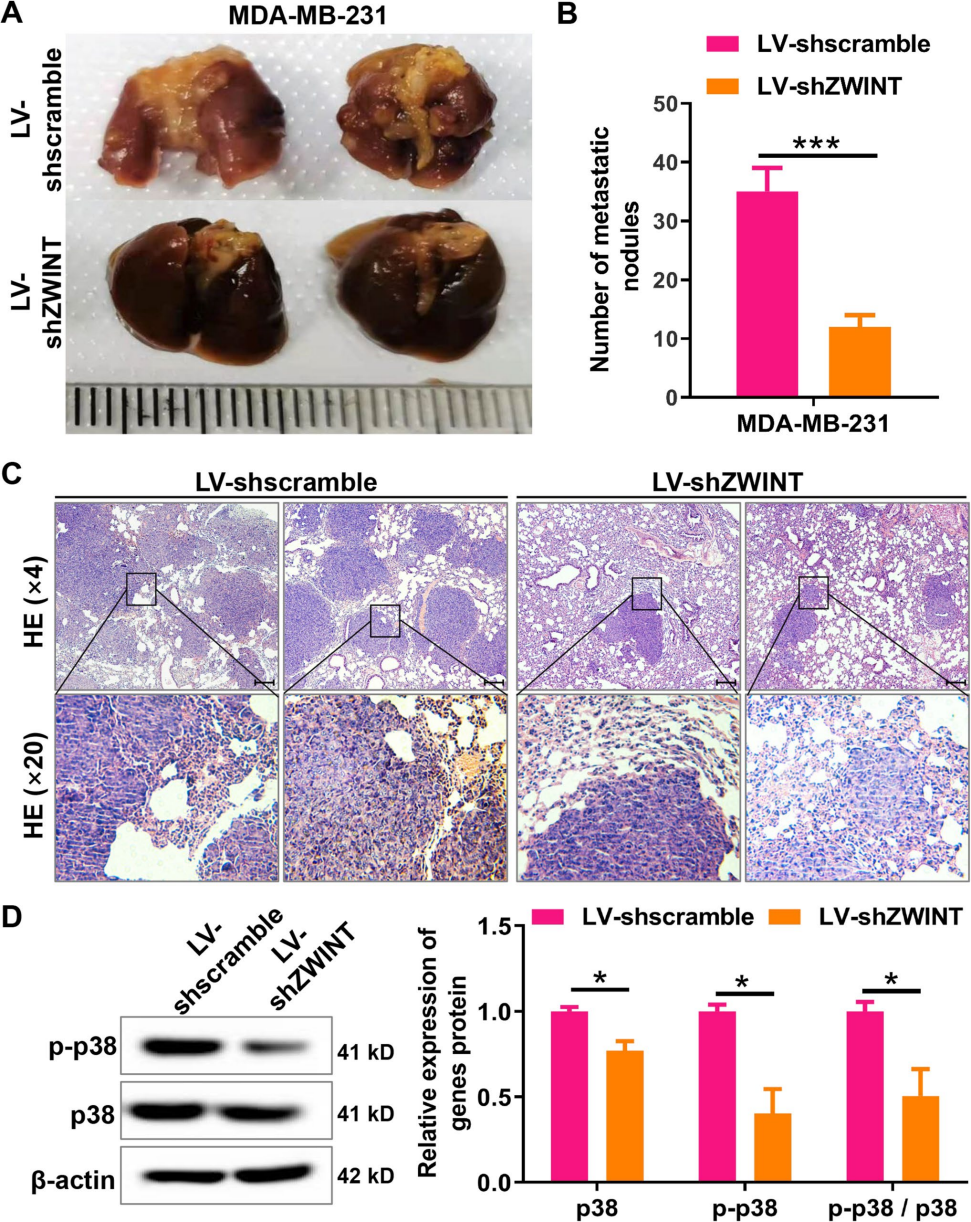
ZWINT knockdown suppresses breast cancer lung metastasis and p38 MAPK pathway in vivo. **A** Representative morphology of lung tissue. **B** Quantification of pulmonary metastatic nodules. **C** HE staining of lung tissue, demonstrating metastatic lesions. Bar = 100 μm, and the black arrow indicates the nodule. **D** The impact of ZWINT knockdown on the protein expression levels of p38 MAPK (p38), phospho-p38 MAPK (p-p38), and the p-p38/p38 ratio in lung metastatic tissues from nude mice was assessed using western blot analysis. *N* = 3–6, *P < 0.05 and ***P < 0.001

To further explore the role of ZWINT in breast cancer metastasis, we established a lung metastasis model in nude mice by injecting MDA-MB-231 cells with ZWINT knockdown into the tail vein. Our results showed that ZWINT knockdown significantly reduced the lung metastasis burden in mice after intravenous injection of MDA-MB-231 cells (Fig. 3A, B). Additionally, HE staining revealed that ZWINT knockdown markedly decreased the number of metastatic lesions in the lung tissue of nude mice **(**Fig. 3C), further confirming that ZWINT knockdown inhibits lung metastasis of breast cancer. The results from western blot revealed that ZWINT knockdown significantly inhibited the protein expression levels of total p38 MAPK and Phospho-p38 MAPK, as well as significantly reduced the Phospho-p38 MAPK/p38 MAPK ratio in vivo (Fig. 3D), suggesting that ZWINT knockdown inhibits the activation of the p38 MAPK signaling pathway in vivo. Collectively, these data suggest that ZWINT may serve as a potential therapeutic target for the treatment of breast cancer lung metastasis.

### miR-495-3p directly targets ZWINT to inhibit its expression

**Fig. 4:**
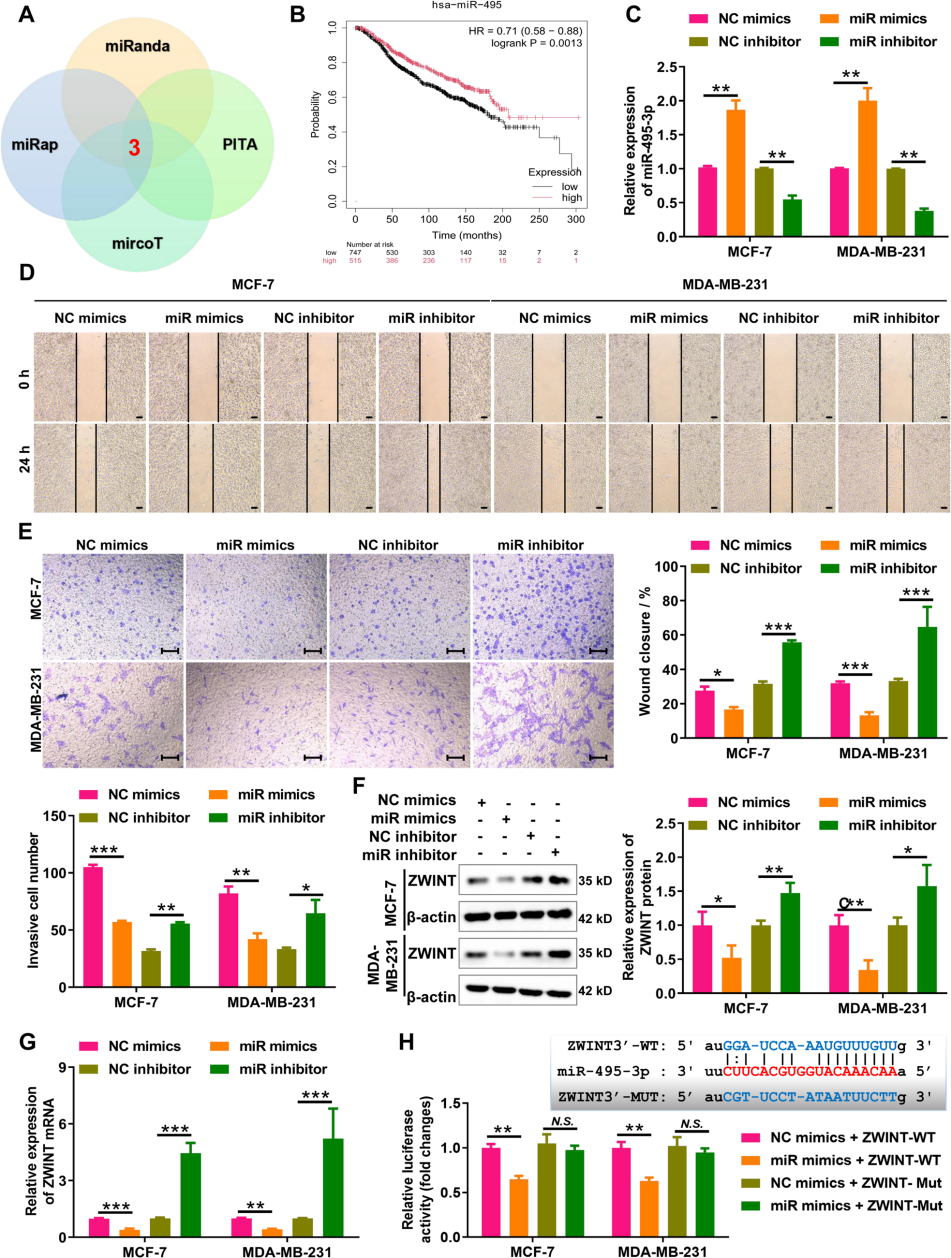
miR-495-3p directly targets ZWINT to inhibit its expression. **A** Three miRNAs (miR-495-3p, miR-124-3p, and miR-506-3p) were predicted to have potential binding sites with ZWINT based on miRanda, miRap, mircoT, and PITA software. **B** Kaplan–Meier plotter showed that breast cancer patients with high expression of miR-495-3p have a high survival rate. **C** miR-495-3p levels in breast cancer cells transfected with mimics/inhibitors or negative controls were quantified by RT-qPCR. **D** Wound-healing assay assessed migration of breast cancer cells transfected with miR-495-3p mimics/inhibitor and their negative control. Bar = 100 μm. **E** Transwell invasion assay evaluated invasion of breast cancer cells transfected with miR-495-3p mimics/inhibitor and their negative control. Bar = 100 μm. **F** RT-qPCR analysis of ZWINT mRNA levels in breast cancer cells transfected with miR-495-3p mimics/inhibitor and their negative control. **G** Western blot analysis of ZWINT protein levels in breast cancer cells transfected with miR-495-3p mimics/inhibitor and their negative control. **H** Luciferase reporter assay was used to analyze the interaction between miR-495-3p and ZWINT. NC, negative control. *miR* miR-495-3p, *WT* wild type, *Mut* mutant type. *N* = 3, ^*N.S.*^*P* > 0.05, *P < 0.05, **P < 0.01, and ***P < 0.001

To elucidate the molecular mechanism underlying ZWINT dysregulation in breast cancer, we used miRanda, miRmap, miRcoT, and PITA software to predict miRNAs that potentially bind to ZWINT. The results indicated that miR-124-3p, miR-495-3p, and miR-506-3p had potential binding sites with ZWINT (Fig. 4A). Analysis of ENCORI online data revealed that only miR-495-3p was significantly negatively correlated with ZWINT in breast cancer, whereas miR-124-3p and miR-506-3p showed no correlation or a positive correlation with ZWINT (Supplementary Fig.  3 A). Kaplan–Meier plotter showed that only high expression of miR-495-3p was negatively correlated with poor prognosis of breast cancer patients (Fig. 4B and Supplementary Fig. 3B). Furthermore, ENCORI data indicated that miR-495-3p was significantly downregulated in breast cancer tissues compared to normal breast tissues (Supplementary Fig.  3 C), and further RT-qPCR results also demonstrated that miR-495-3p expression was significantly lower in MCF-7 and MDA-MB-231 cells compared with MCF-10A (Supplementary Fig. 3D). Next, to confirm the role of miR-495-3p in breast cancer cell migration and invasion, we transfected the breast cancer cells with miR-495-3p mimics and miR-495-3p inhibitor, respectively. The results of RT-qPCR analysis demonstrated that transfection of miR-495-3p mimics in breast cancer cells significantly increased the expression of miR-495-3p, and transfection of miR-495-3p inhibitor could significantly reduce the expression of miR-495-3p in breast cancer cells (Fig. 4C). The results of wound-healing assay showed that miR-495-3p knockdown significantly promoted migration and invasion of MCF-7 and MDA-MB-231 cells, whereas miR-495-3p overexpression significantly inhibited migration and invasion of MCF-7 and MDA-MB-231 cells (Fig. 4D). The results of Transwell invasion assay demonstrated that miR-495-3p knockdown significantly promoted cell invasion of MCF-7 and MDA-MB-231 cells, whereas miR-495-3p overexpression significantly inhibited promoted cell invasion of MCF-7 and MDA-MB-231 cells (Fig. 4E). Altogether, the above data suggest that miR-495-3p plays a protective role in breast cancer migration and invasion.

Subsequently, we analyzed the regulatory relationship between miR-495-3p and ZWINT. The results of RT-qPCR and western blot analysis showed that overexpression of miR-495-3p significantly inhibited ZWINT expression at both the mRNA and protein levels in MCF-7 and MDA-MB-231 cells, while miR-495-3p knockdown had the opposite effect (Fig. 4F, G), indicating that miR-495-3p negative regulates ZWINT expression in breast cancer. Further luciferase reporter experiments showed that that overexpression of miR-495-3p significantly inhibited the luciferase activity of ZWINT-WT but not ZWINT-Mut in MCF-7 and MDA-MB-231 cells (Fig. 4H). In conclusion, miR-495-3p, which inhibits breast cancer cell migration and invasion, inhibits ZWINT inhibition by sponge adsorption of ZWINT.

### miR-495-3p inhibits the migration and invasion of breast cancer cells by regulating ZWINT-mediated p38 MAPK pathway

**Fig. 5:**
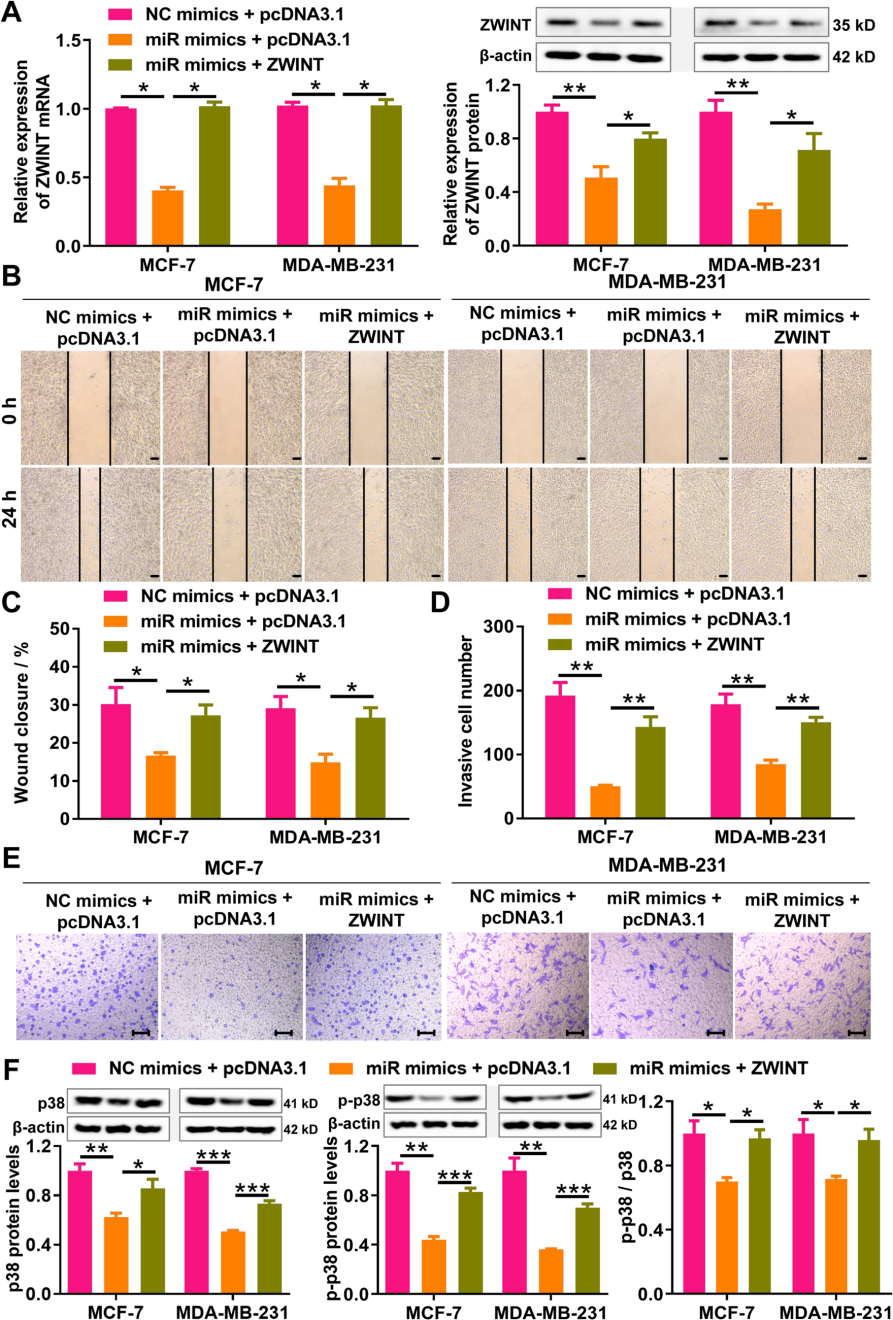
Overexpression of miR-495-3p inhibits breast cancer cell migration and invasion and suppresses the p38 MAPK pathway by downregulating ZWINT. **A** RT-PCR and western blot assessed ZWINT mRNA and protein levels in breast cancer cells transfected with miR-495-3p alone or combined with ZWINT. **B**, **C** A wound-healing assay assessed the migration of breast cancer cells transfected with miR-495-3p alone or combined with ZWINT. Bar = 100 μm. **D**, **E** Transwell invasion assay was used to detect the invasion ability of breast cancer cells transfected with miR-495-3p alone or in combination with ZWINT. Bar = 100 μm. **F** Western blot analysis of p38 MAPK (p38), phospho-p38 MAPK (p-p38), and the p-p38/p38 ratio was performed in breast cancer cells transfected with miR-495-3p alone or co-transfected with ZWINT. NC, negative control. *miR* miR-495-3p. *N* = 3, *P < 0.05, **P < 0.01, and ***P < 0.001

To explore whether miR-495-3p affects breast cancer cell migration, invasion, and the p38 MAPK pathway via ZWINT regulation, we performed reversion experiments in MCF-7 and MDA-MB-231 cells. RT-qPCR and western blot data indicated that transfecting breast cancer cells with the ZWINT overexpression plasmid significantly reversed the suppressive effect of miR-495-3p on ZWINT expression (Fig. 5A). Wound-healing assay subsequently revealed that miR-495-3p overexpression substantially inhibited cell migration and invasion in both MCF-7 and MDA-MB-231 cells, an effect that was markedly reduced by ZWINT overexpression (Fig. 5B, C). Transwell invasion assay revealed that miR-495-3p overexpression substantially inhibited cell migration and invasion in both MCF-7 and MDA-MB-231 cells, and the effect was markedly reduced by ZWINT overexpression (Fig. 5D, E). Furthermore, western blot analysis showed that ZWINT overexpression restored the protein expression levels of p38 MAPK and Phospho-p38 MAPK, as well as the Phospho-p38 MAPK/p38 MAPK ratio, which had been suppressed by miR-495-3p overexpression (Fig. 5F). Collectively, these results suggest that miR-495-3p overexpression inhibits breast cancer cell migration, invasion, and p38 MAPK pathway activation by down-regulating ZWINT.

## Discussion

Metastasis remains a leading contributor to mortality in breast cancer patients, underscoring the urgent need to elucidate its molecular drivers [25–27]. Our previous results have shown that high expression of ZWINT in breast cancer tissues is closely related to DMFS of breast cancer patients, suggesting that ZWINT may be involved in the invasion and metastasis of breast cancer [16]. However, the role of ZWINT in metastatic dissemination of breast cancer remains unexplored. Our study bridges this critical knowledge gap by demonstrating that ZWINT silencing suppresses breast cancer cell migration and invasion in vitro and attenuates lung metastasis in vivo through inhibition of the p38 MAPK pathway. Mechanistically, we identify miR-495-3p as a direct post-transcriptional regulator of ZWINT, establishing a novel miR-495-3p/ZWINT/p38 MAPK axis governing metastatic behavior.

The oncogenic function of ZWINT exhibits significant tissue context dependency. In hepatocellular carcinoma, ZWINT emerges as a promising therapeutic biomarker associated with the tumor immune microenvironment, and its elevated expression correlates with aggressive clinicopathological features, including lymph-node metastasis and vascular invasion [28, 29]. Melanoma studies demonstrate that ZWINT promotes tumor cell proliferation and migration by regulating c-Myc expression, highlighting its dual role in driving these malignant phenotypes [30]. In breast cancer, ZWINT—directly targeted and suppressed by miR-204—forms a positive feedback loop with CDK1 to drive G1/S phase transition and promote proliferation [13, 14]. However, the role and regulatory mechanisms of ZWINT within the breast cancer metastatic microenvironment remain undefined. This study provides compelling evidence for ZWINT's involvement in breast cancer invasion and metastasis. A series of functional experiments in this study demonstrated that ZWINT knockdown significantly inhibited breast cancer cell migration and invasion in vitro, and suppressed lung metastasis in vivo. Importantly, we extend these observations by linking ZWINT to metastasis-specific processes in breast cancer. The association between high ZWINT levels and reduced DMFS in clinical cohorts [16] further supports its potential as a prognostic biomarker and therapeutic target. While our findings establish a critical role for ZWINT in metastatic dissemination, we acknowledge a limitation of the experimental approach. The tail vein injection model efficiently isolates and demonstrates ZWINT's function in the lung colonization step of metastasis but does not recapitulate spontaneous metastasis from orthotopic sites. Future studies employing orthotopic models will be essential to validate these findings within the context of the complete metastatic cascade. Collectively, our results identify ZWINT as a key driver of breast cancer metastasis and a promising therapeutic target.

The p38 MAPK signaling pathway is critical to tumorigenesis and metastasis, with its activation observed across a wide range of tumor types [31–33]. However, the role of p38 MAPK signaling pathway in tumorigenesis and metastasis of breast cancer remains controversial. Some studies have demonstrated that p38 MAPK activation promotes breast cancer progression [34–38]. On the contrary, other studies have shown that activation of p38 MAPK signaling pathway can inhibit breast cancer growth and metastasis [39, 40]. In this study, we found that ZWINT knockdown reduced the protein expression levels of p38 MAPK and its phosphorylation, as well as the Phospho-p38 MAPK/p38 MAPK ratio, indicating that ZWINT knockdown inhibited p38 MAPK pathway activation in breast cancer. Furthermore, our data showed that Anisomycin, an activator of the p38 MAPK pathway, significantly reversed the inhibitory effects of ZWINT knockdown on the migration and invasion of breast cancer cells, suggesting that ZWINT silencing suppresses breast cancer cell invasion and metastasis by blocking p38 MAPK pathway activation. Consistent with our findings, Fang et al. [41] and Wang et al. [42] also revealed that activation of p38 MAPK signaling promoted breast cancer cell proliferation, invasion, and migration. Collectively, these studies highlight the p38 MAPK signaling pathway as a key regulator of breast cancer cell invasion and metastasis, and suggest that targeted inhibition of its activation could be an effective strategy for controlling breast cancer tumorigenesis and metastasis. Notably, the conflicting roles of p38 MAPK may stem from isoform-specific effects (p38α vs. p38β) or differential activation thresholds in distinct molecular subtypes—a nuance requiring further investigation using isoform-selective inhibitors and genetically engineered models.

The regulatory interplay between miRNAs and their mRNA targets has emerged as a critical mechanism governing tumor progression, including breast cancer metastasis [43]. Our findings reveal that diminished miR-495-3p expression in breast cancer cells disrupts its direct targeting of ZWINT through 3′UTR binding, leading to ZWINT overexpression—a key driver of metastatic behavior. Importantly, *ZWINT* is co-regulated by a network of tumor-suppressive miRNAs across cancer types. For example, Zhang et al. demonstrated that miR-204 directly binds the *ZWINT* 3′-UTR to suppress proliferation in breast cancer [13]. While miR-204 target *ZWINT*, our functional assays establish miR-495-3p as the dominant upstream suppressor specifically governing metastatic progression in breast cancer models. we note other miRNAs with similar roles: Additionally, we note that miR-508-3p may function as a tumor suppressor in lung adenocarcinoma by targeting *ZWINT*, where its downregulation accelerates suppression of tumor cell proliferation, apoptosis inhibition, and invasion, thereby inhibiting the progression of lung adenocarcinoma [44]. This finding further highlights the network complexity and context dependency of *ZWINT* regulation.

Previous studies present conflicting evidence regarding miR-495-3p's function in breast cancer. Reports suggesting oncogenic activity cite its capacity to downregulate E-cadherin and REDD1 to promote tumor growth [45], its elevated expression in clinical specimens correlating with migration [46], and its pro-tumorigenic effects through Bmi-1 regulation [47]. However, these studies predominantly focused on primary tumor growth rather than metastatic dissemination. In contrast, our metastasis-centric approach demonstrates that miR-495-3p overexpression significantly impedes migration and invasion—a finding corroborated by independent studies showing its suppression of STAT3-mediated proliferation [48] and inhibition of malignant phenotypes through undefined targets [49]. This functional dichotomy likely stems from three factors, such as hormone receptor status or molecular subtype may dictate miRNA target preference, early tumorigenesis and late metastasis may involve distinct miRNA regulatory networks, and beyond ZWINT, miR-495-3p may concurrently regulate multiple metastasis-relevant genes. Notably, we identified p38 MAPK signaling as the critical nodal point linking miR-495-3p to metastasis suppression. Through ZWINT downregulation, miR-495-3p attenuates p38 phosphorylation, subsequently inhibiting downstream effectors of cytoskeletal remodeling and extracellular matrix degradation. This mechanism aligns with miR-495-3p's reported p38-activating role in osteoblast differentiation [50], suggesting tissue-specific pathway modulation, and this phenomenon requiring further investigation using organotropic metastasis models.

## Limitations

While our in vitro findings robustly establish the role of the miR-495-3p/ZWINT/p38 MAPK axis in suppressing metastasis-associated phenotypes, two critical translational gaps remain. First, although the data indicate that miR-495-3p acts as an upstream regulator of ZWINT, the functional significance of this axis in vivo has not yet been established. Studies utilizing miR-495-3p agomirs or antagomirs in animal models will be essential to validate the physiological relevance of this pathway in breast cancer metastasis. Second, the clinical correlations between miR-495-3p levels, ZWINT/p38 MAPK pathway, and metastatic relapse remain unestablished. Further investigations should employ orthotopic or experimental metastasis models to evaluate the efficacy of miR-495-3p-based interventions in suppressing metastatic dissemination to sites such as lung and bone. Complementary in vivo studies of ZWINT/p38 MAPK pathway modulation, along with spatial transcriptomic profiling of patient-matched primary and metastatic tissues, will be essential to delineate the dynamics of pathway activation during metastatic progression.

## Conclusions

Our data suggest that ZWINT is a potential therapeutic target for breast cancer invasion and metastasis. Most importantly, our results revealed that overexpression of miR-495-3p inhibited breast cancer cell invasion and metastasis by blocking ZWINT-mediated p38 MAPK pathway activation. These results suggest that the miR-495-3p/Z WINT axis is a novel target for suppressing invasion and metastasis of breast cancer.

## Supplementary Information

Below is the link to the electronic supplementary material.

Supplementary file1 Figure S1 The average correlation between ZWINT and the biological functions of breast cancer cells was evaluated through the CancerSEA database. (A) Heat map of correlation between ZWINT and 14 cell functions. The darker the red indicate the stronger the correlation and the darker the blue indicate the weaker the correlation. (B) Breast cancer cell functions significantly associated with ZWINT in EXP0052 data analysis. (C) Breast cancer cell functions significantly associated with ZWINT in EXP0053 data analysis. (D) Breast cancer cell functions significantly associated with ZWINT in EXP0054 data analysis. (E) Breast cancer cell functions shown to be significantly associated with ZWINT in EXP0055 data analysis (TIF 15802 KB)

Supplementary file2 Figure S2 (A) RT-PCR was used to detect the mRNA expression level of ZWINT in MCF-7 and MDA-MB-231 cells transfected with shZWINT or its negative control. (B) Western blot was used to detect the protein expression level of ZWINT in MCF-7 and MDA-MB-231 cells transfected with shZWINT or its negative control. N = 3, N.S.P > 0.05, *P < 0.05, **P < 0.01, and ***P < 0.001 (TIF 11614 KB)

Supplementary file3 Figure S3 (A) ENCORI online data was used to analyze the correlation between ZWINT and miR-495-3p, miR-124-3p and miR-506-3p in breast cancer. (B) Kaplan Meier plotter showed the relationship between the expression levels of miR-124-3p/miR-506-3p and the survival rate of breast cancer patients. (C) ENCORI online data was used to analyze the expression level of miR-495-3p in normal breast tissues and breast cancer tissues. (D) RT-PCR was used to detect the miR-495-3p expression levels in MCF-10A, MCF-7, and MDA-MB-231 cells. N = 3, *P < 0.05 (TIF 19149 KB)

Supplementary file4 (PDF 1243 KB)

Supplementary file5 (DOCX 16 KB)

## Data Availability

The datasets analyzed during the current study are available from the corresponding authors on reasonable requests.
