# Supplementary figures and images for: ZWINT down-regulated by miR-495-3p inhibited lung metastasis of breast cancer by blocking p38 MAPK signaling pathway activation
Source: Hum Cell. 2025 Oct 4;38(6):170. doi: 10.1007/s13577-025-01301-z (PMC12496286; doi:10.1007/s13577-025-01301-z)

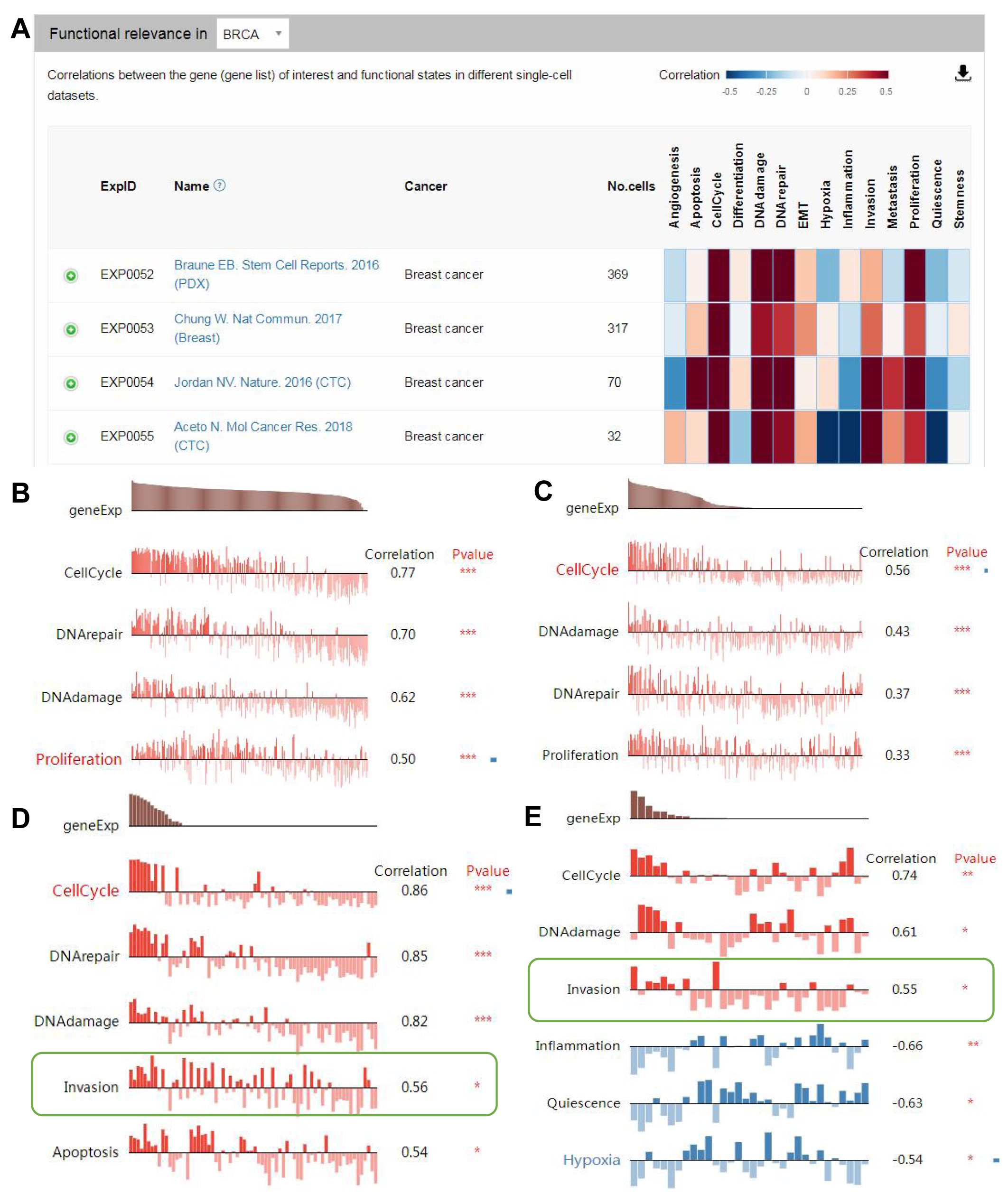

Supplement: Supplementary file 1 — Supplementary file1 Figure S1 The average correlation between ZWINT and the biological functions of breast cancer cells was evaluated through the CancerSEA database. (A) Heat map of correlation between ZWINT and 14 cell functions. The darker the red indicate the stronger the correlation and the darker the blue indicate the weaker the correlation. (B) Breast cancer cell functions significantly associated with ZWINT in EXP0052 data analysis. (C) Breast cancer cell functions significantly associated with ZWINT in EXP0053 data analysis. (D) Breast cancer cell functions significantly associated with ZWINT in EXP0054 data analysis. (E) Breast cancer cell functions shown to be significantly associated with ZWINT in EXP0055 data analysis (TIF 15802 KB) [file 13577_2025_1301_MOESM1_ESM.tif]

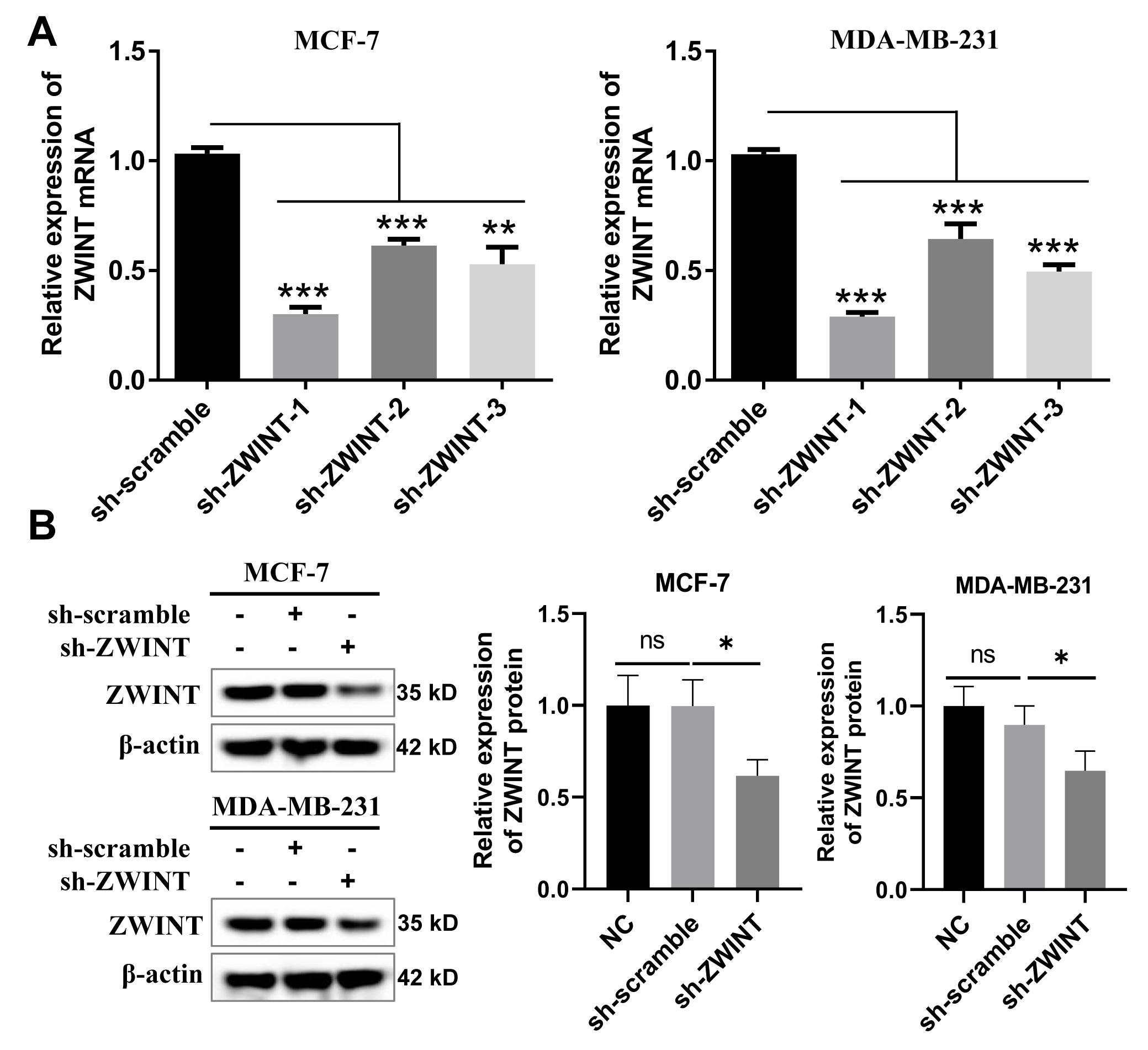

Supplement: Supplementary file 2 — Supplementary file2 Figure S2 (A) RT-PCR was used to detect the mRNA expression level of ZWINT in MCF-7 and MDA-MB-231 cells transfected with shZWINT or its negative control. (B) Western blot was used to detect the protein expression level of ZWINT in MCF-7 and MDA-MB-231 cells transfected with shZWINT or its negative control. N = 3, N.S.P > 0.05, *P < 0.05, **P < 0.01, and ***P < 0.001 (TIF 11614 KB) [file 13577_2025_1301_MOESM2_ESM.tif]

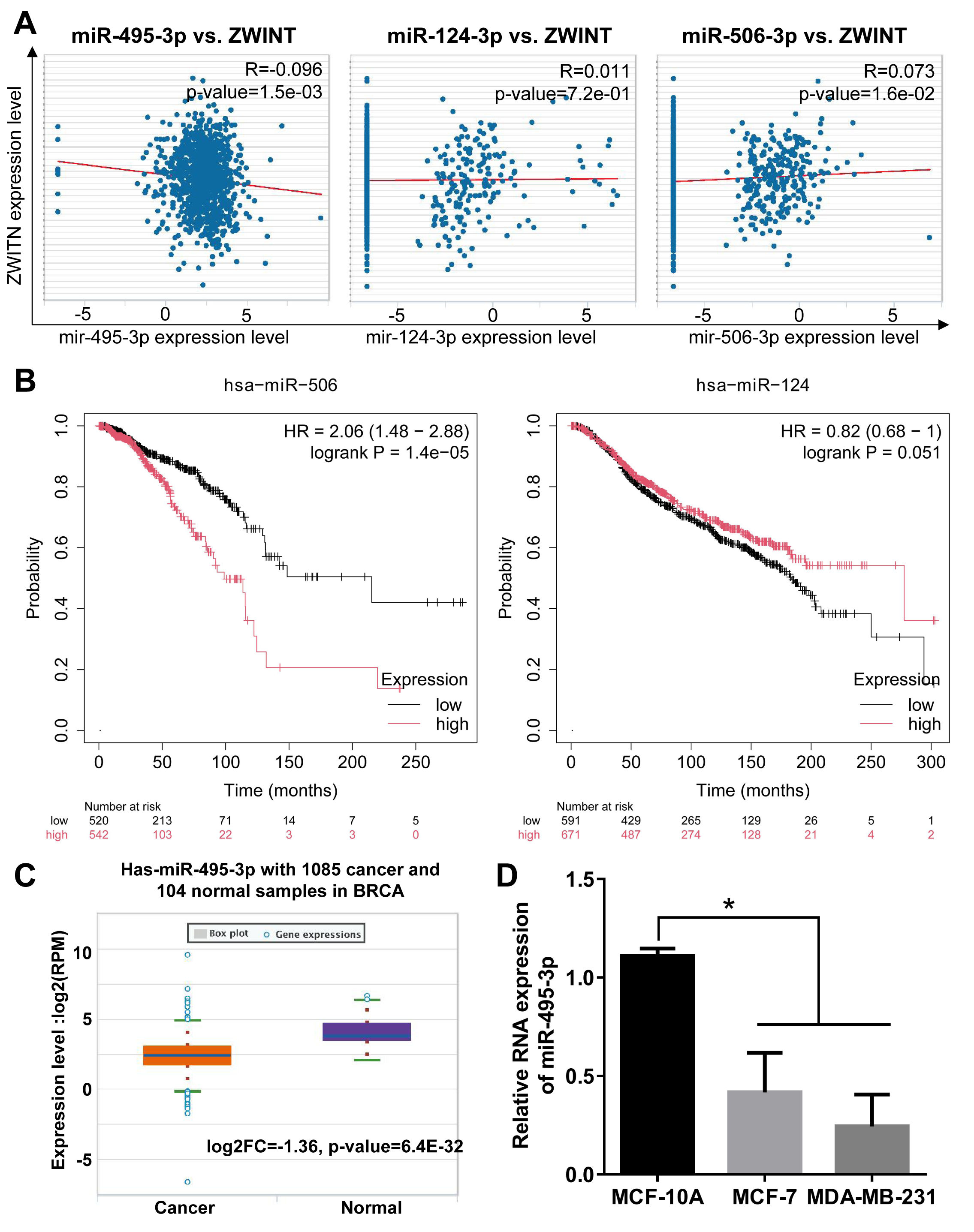

Supplement: Supplementary file 3 — Supplementary file3 Figure S3 (A) ENCORI online data was used to analyze the correlation between ZWINT and miR-495-3p, miR-124-3p and miR-506-3p in breast cancer. (B) Kaplan Meier plotter showed the relationship between the expression levels of miR-124-3p/miR-506-3p and the survival rate of breast cancer patients. (C) ENCORI online data was used to analyze the expression level of miR-495-3p in normal breast tissues and breast cancer tissues. (D) RT-PCR was used to detect the miR-495-3p expression levels in MCF-10A, MCF-7, and MDA-MB-231 cells. N = 3, *P < 0.05 (TIF 19149 KB) [file 13577_2025_1301_MOESM3_ESM.tif]
