# Supplementary material for: ZWINT down-regulated by miR-495-3p inhibited lung metastasis of breast cancer by blocking p38 MAPK signaling pathway activation
Source: Hum Cell. 2025 Oct 4;38(6):170. doi: 10.1007/s13577-025-01301-z (PMC12496286; doi:10.1007/s13577-025-01301-z)
Supplement: Supplementary file 5 — Supplementary file5 (DOCX 16 KB) [file 13577_2025_1301_MOESM5_ESM.docx]

**Supplemental Table**

**Supplemental Table 1** Sequences of shRNA, miR-495-3p mimics, miR-495-3p inhibitor, and their control negative.

| Name | Sequence |
| --- | --- |
| ZWINT shRNA#1  (sh-ZWINT-1) | CCGGCAGAGAATCTTCCAGATGATACTCGAGTATCATCTGGAAGATTCTCTGTTTTTTG |
| ZWINT shRNA#2  (sh-ZWINT-2) | CCGGGCCAAGATCCTGGTTGAGTTTCTCGAGAAACTCAACCAGGATCTTGGCTTTTTTG |
| ZWINT shRNA#3  (sh-ZWINT-3) | CCGGGTTTCAGAAACTTGGAAACCTCTCGAGAGGTTTCCAAGTTTCTGAAACTTTTTTG |
| scramble shRNA  (sh-scramble) | CCGGTCCTAAGGTTAAGTCGCCCTCGCTCGAGCGAGGGCGACTTAACCTTAGGTTTTTGAATT |
| NC mimics | CUCGGGCCAUCCCAGCCCACUU |
| miR-495-3p mimics | UUCUUCACGUGGUACAAACAAA |
| NC inhibitor | AAGUGGGCUGGGAUGGCCCGAG |
| miR-495-3p inhibitor | UUUGUUUGUACCACGUGAAGAA |

**Supplemental Table 2** Sequences of mRNA and miRNA primers.

| **Name** | **Primer Sequence** |
| --- | --- |
| ZWINT | Forward primer: GCAGCTACAACAGGAGAAGC |
|  | Reverse primer: TCCCTGTCTTACGCTCCCTC |
| GAPDH | Forward primer: CTCCTCTGACTTCAACAGCGAC |
|  | Reverse primer: TAGAAGATGAAAAGAGTTGTCAGGG |
| miR-495-3p | RT primer: GTCGTATCCAGTGCGTGTCGTGGAGTCGG  CAATTGCACTGGATACGACAAGAAGTG |
|  | Forward primer: GGCGAGAAACAAACATGGTG |
|  | Reverse primer: GTCGTATCCAGTGCGTGTC |
| U6 | RT primer: AAAATATGGAACGCTTCACGAATTTG |
|  | Forward primer: CTCGCTTCGGCAGCACATATACT |
|  | Reverse primer: ACGCTTCACGAATTTGCGTGTC |
