# Supplementary material for: ZWINT down-regulated by miR-495-3p inhibited lung metastasis of breast cancer by blocking p38 MAPK signaling pathway activation
Source: Hum Cell. 2025 Oct 4;38(6):170. doi: 10.1007/s13577-025-01301-z (PMC12496286; doi:10.1007/s13577-025-01301-z)
Supplement: Supplementary file 4 — Supplementary file4 (PDF 1243 KB) [file 13577_2025_1301_MOESM4_ESM.pdf]

Dear editor and reviewers

The band order of the uncropped blot images is the same as that in the corresponding figures within the manuscript.

Figure 1A

ZWINT (35kD)

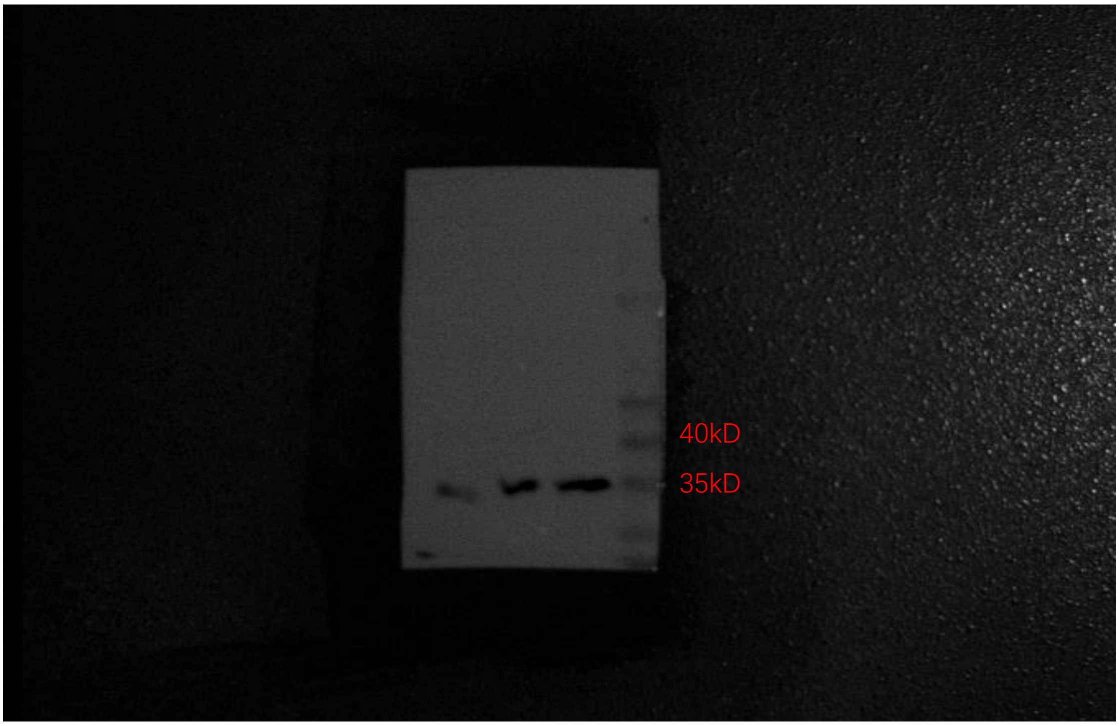

β-actin(42kD)

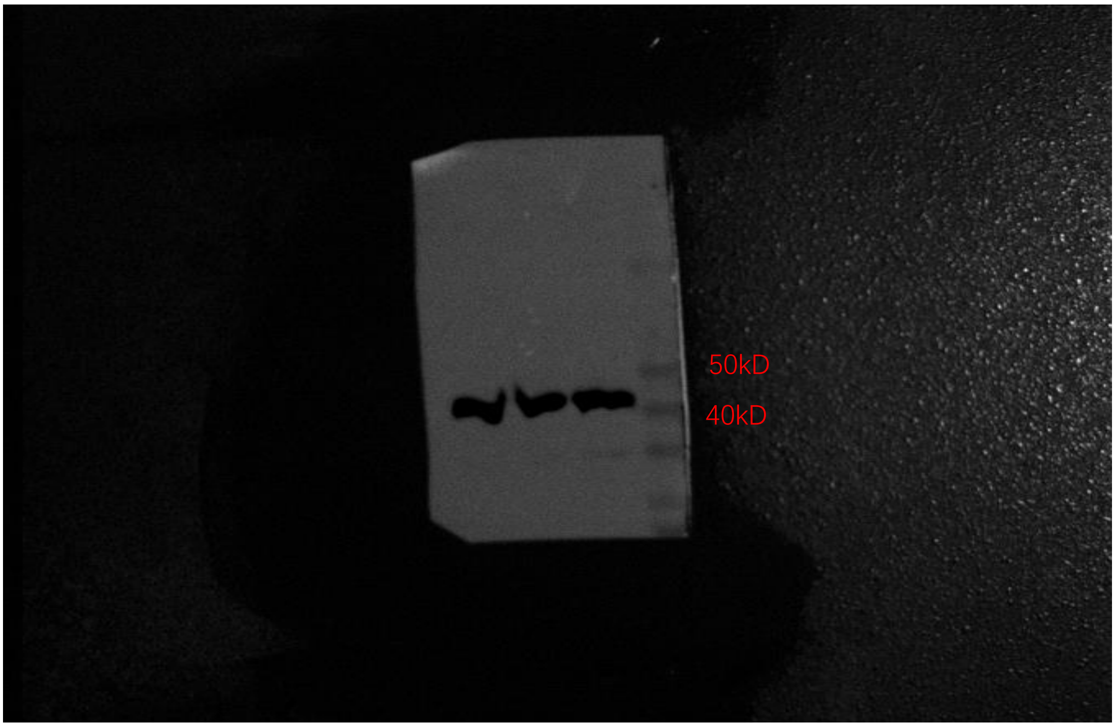

Figure 2A

MCF-7

p-p38 (41kD)

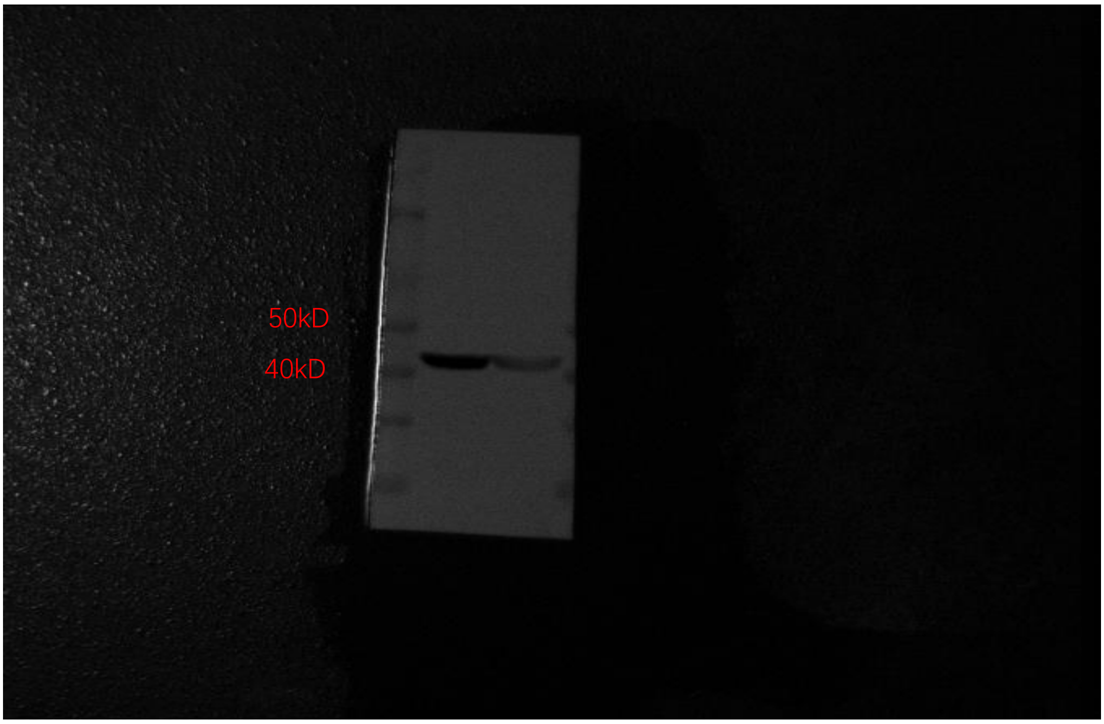

p38 (41kD)

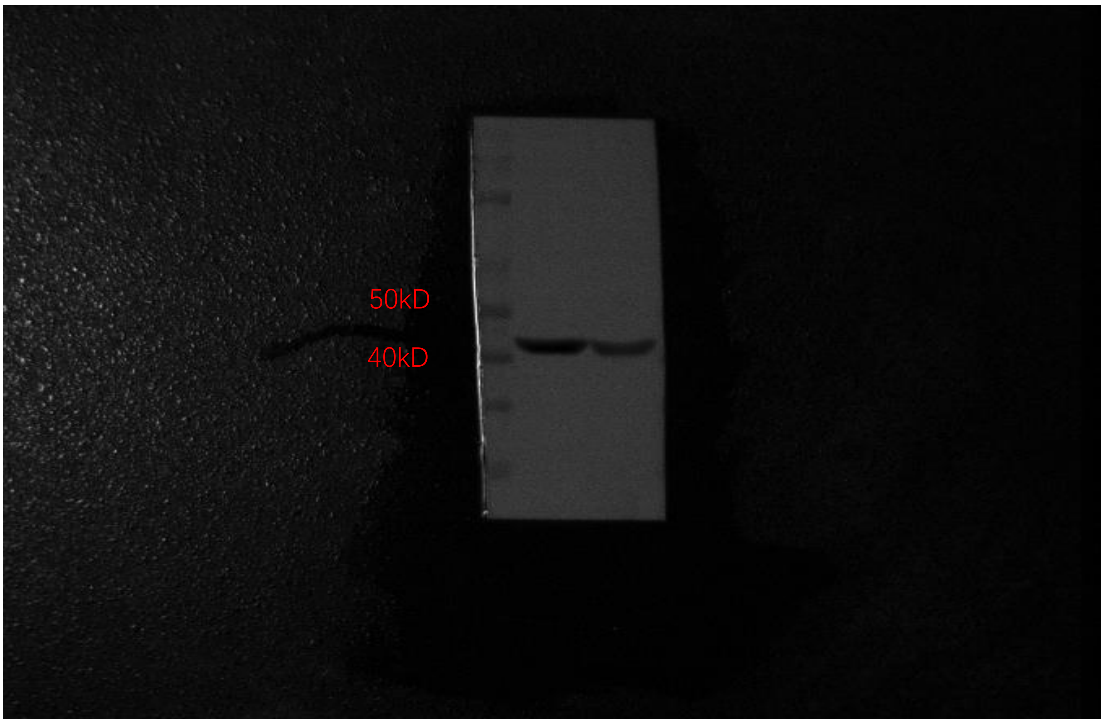

β-actin(42kD)

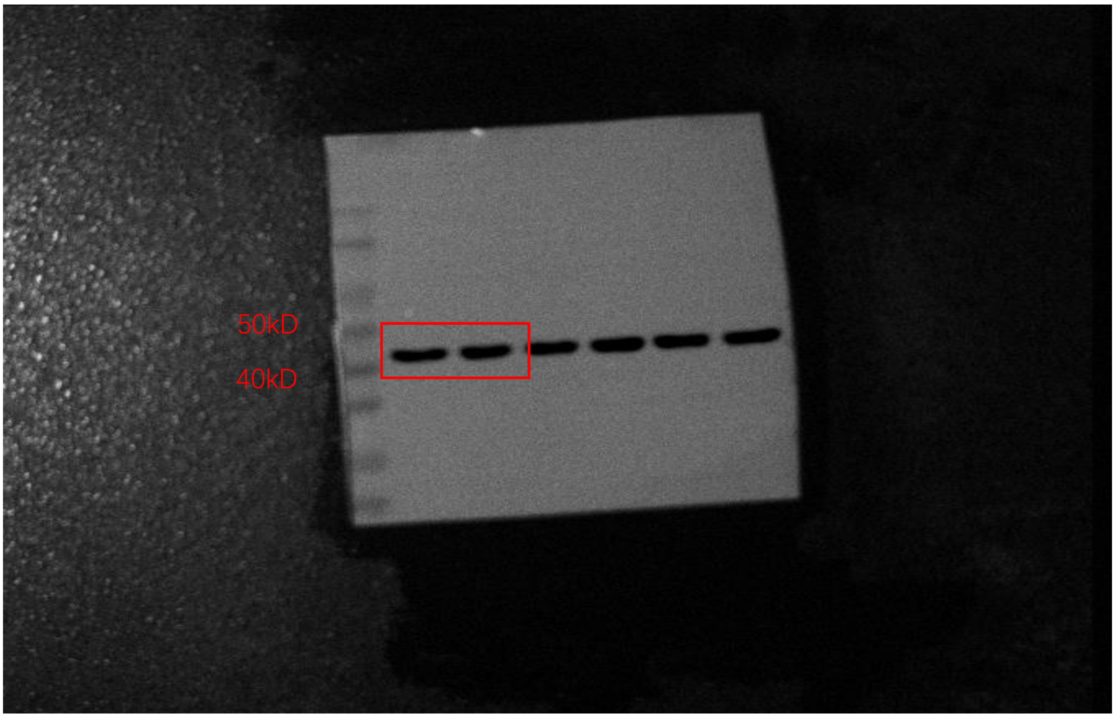

MDA-MB-231
p-p38 (41kD)

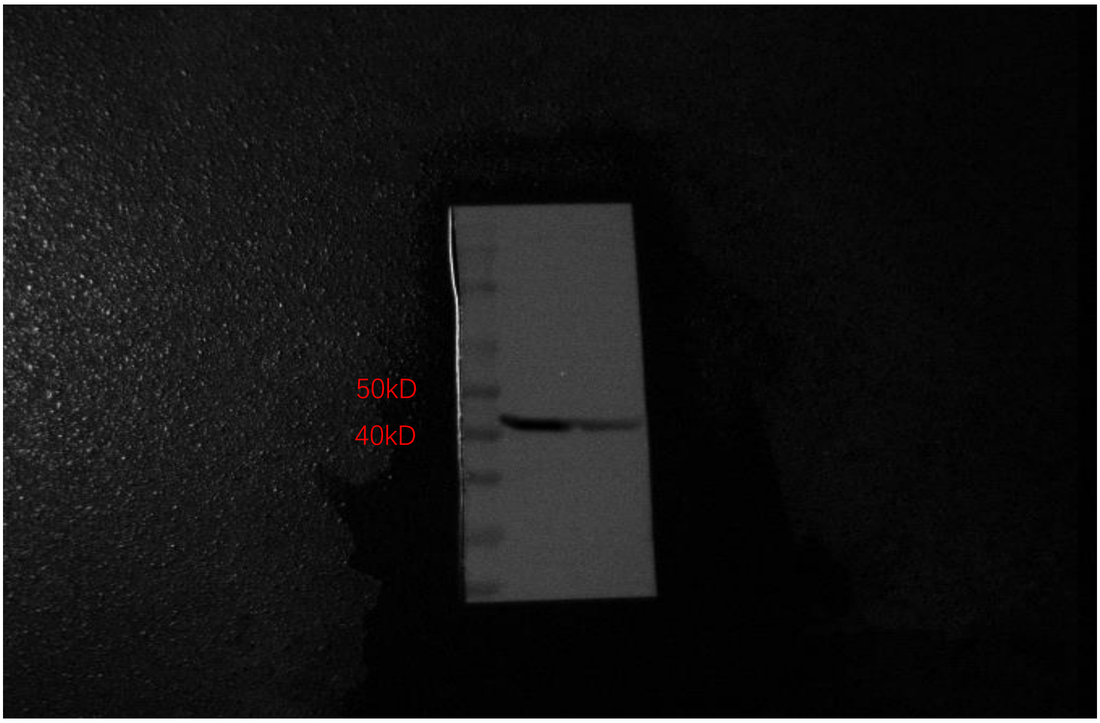

p38 (41kD)

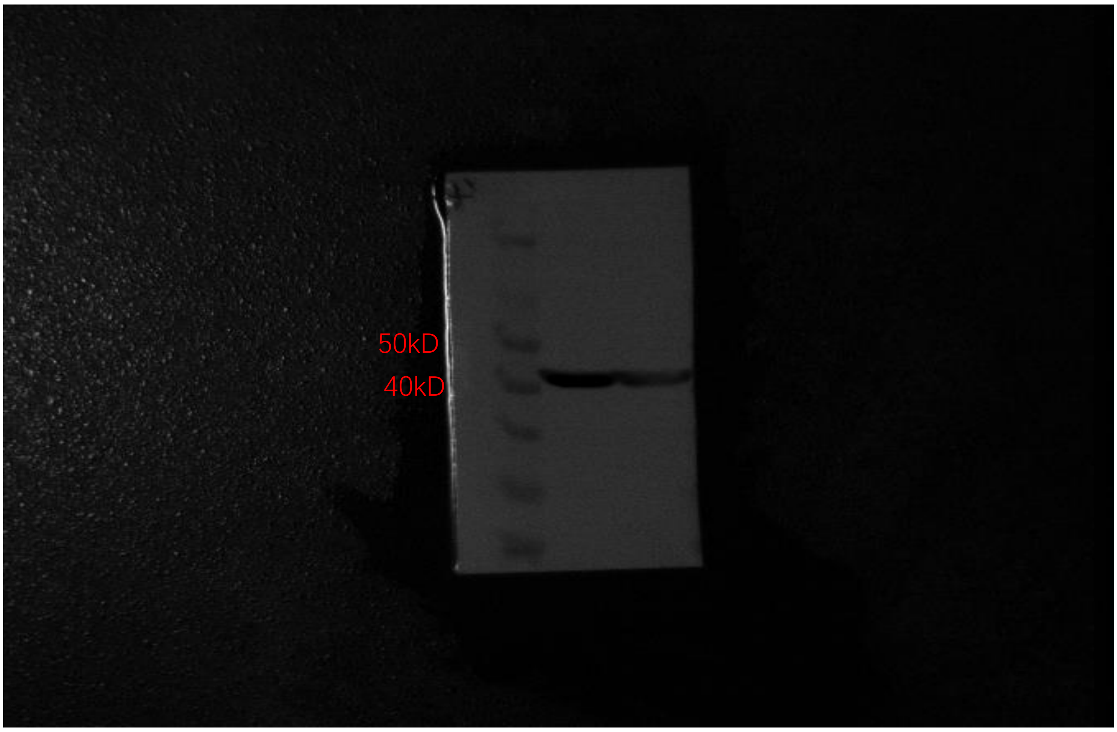

β-actin(42kD)

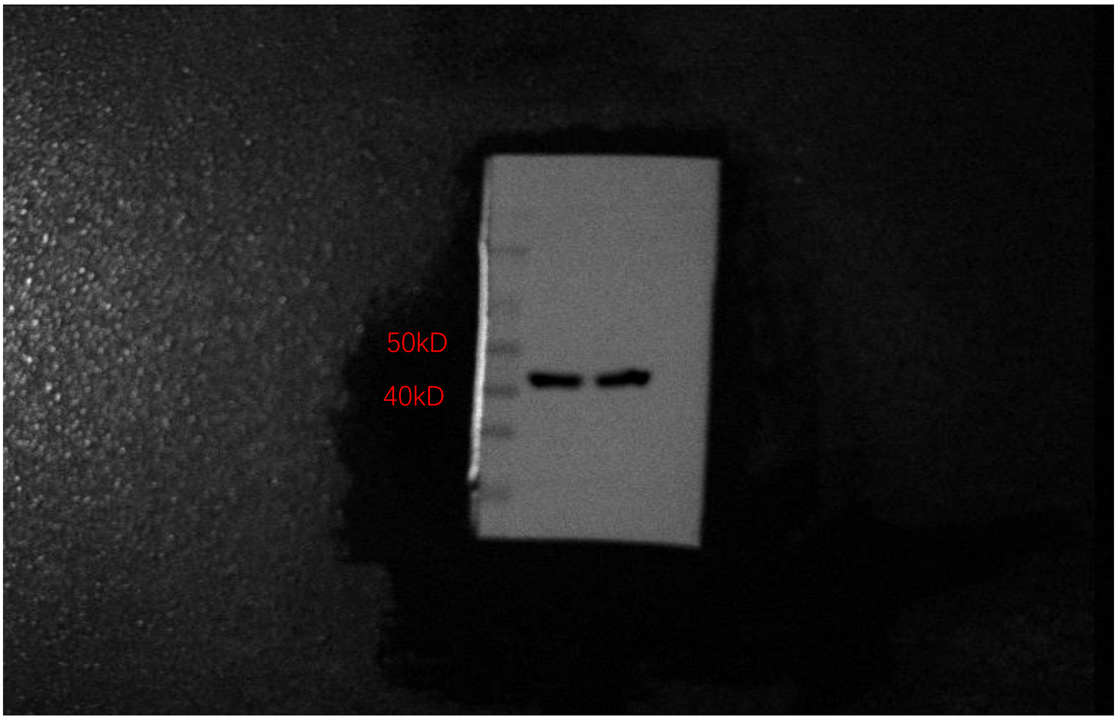

Figure 3D

p-p38 (41kD)

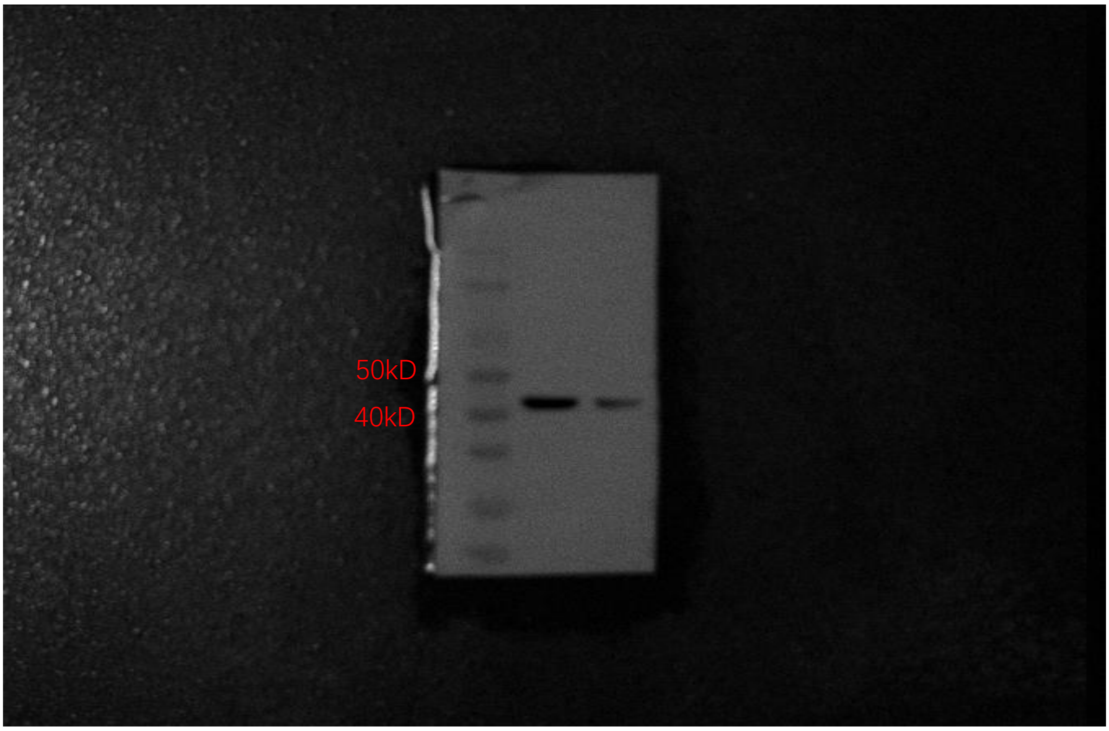

p38 (41kD)

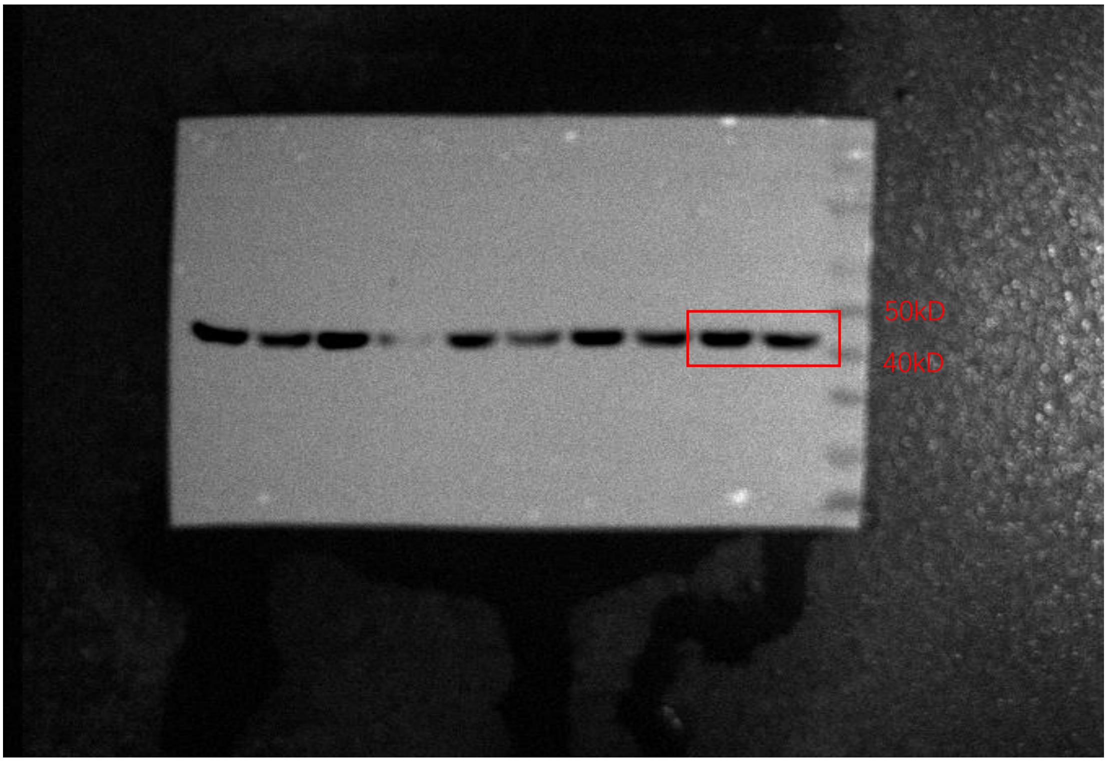

β-actin(42kD)

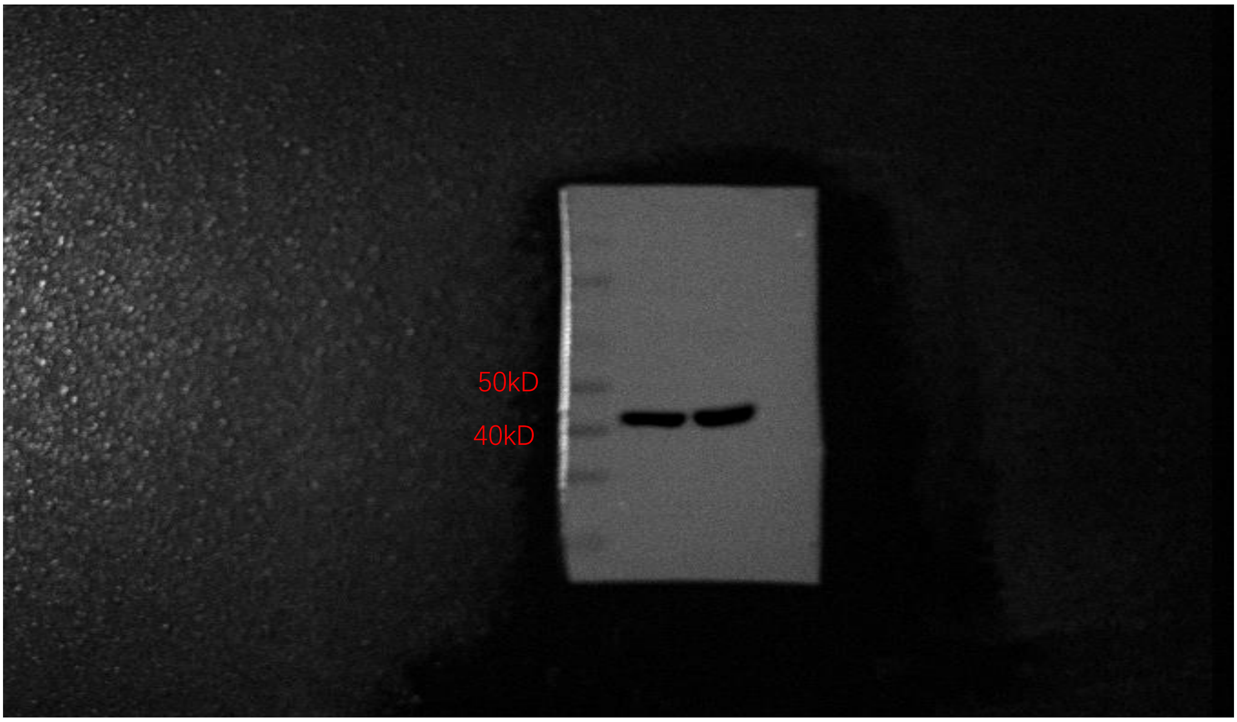

Figure 4F

MCF-7

ZWINT (35kD)

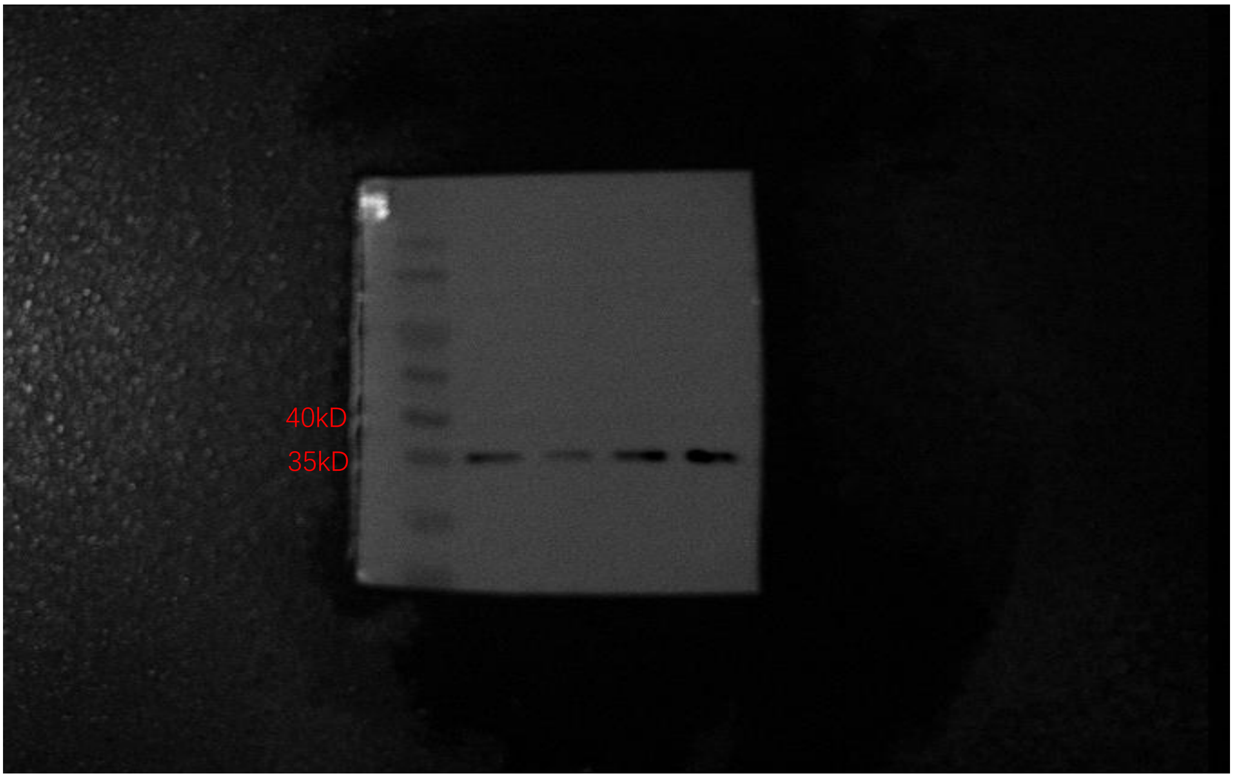

β-actin(42kD)

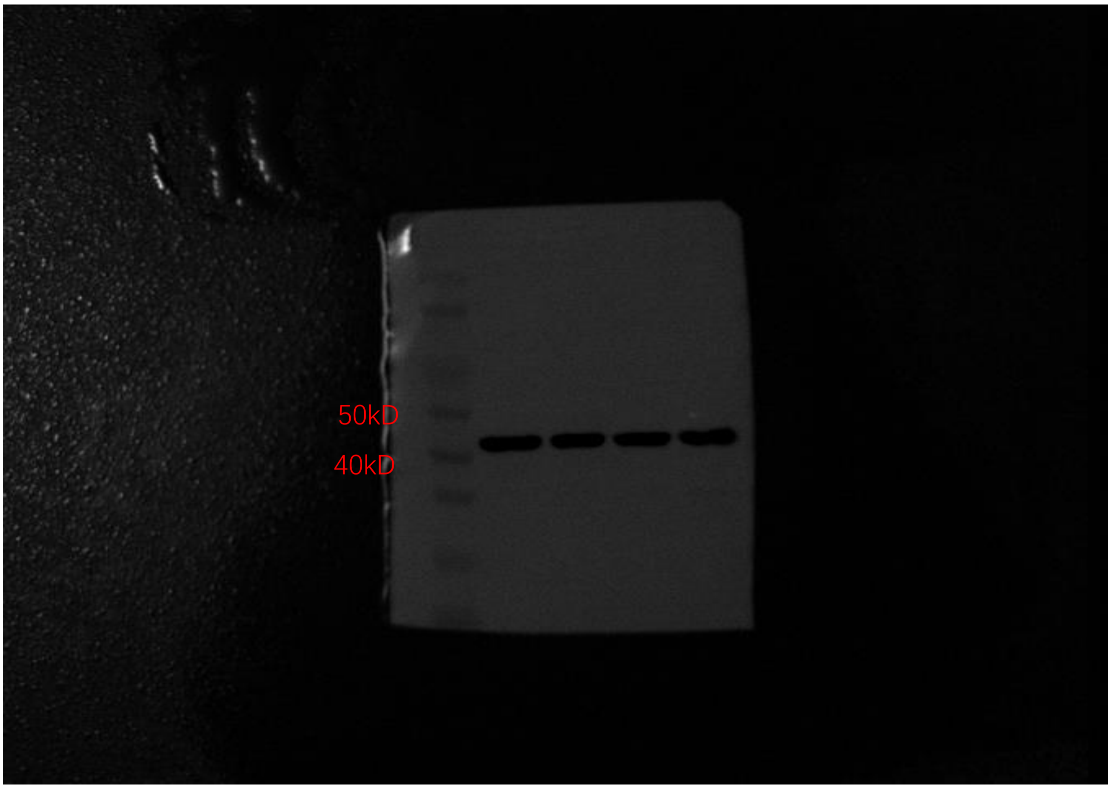

MDA-MB-231
ZWINT (35kD)

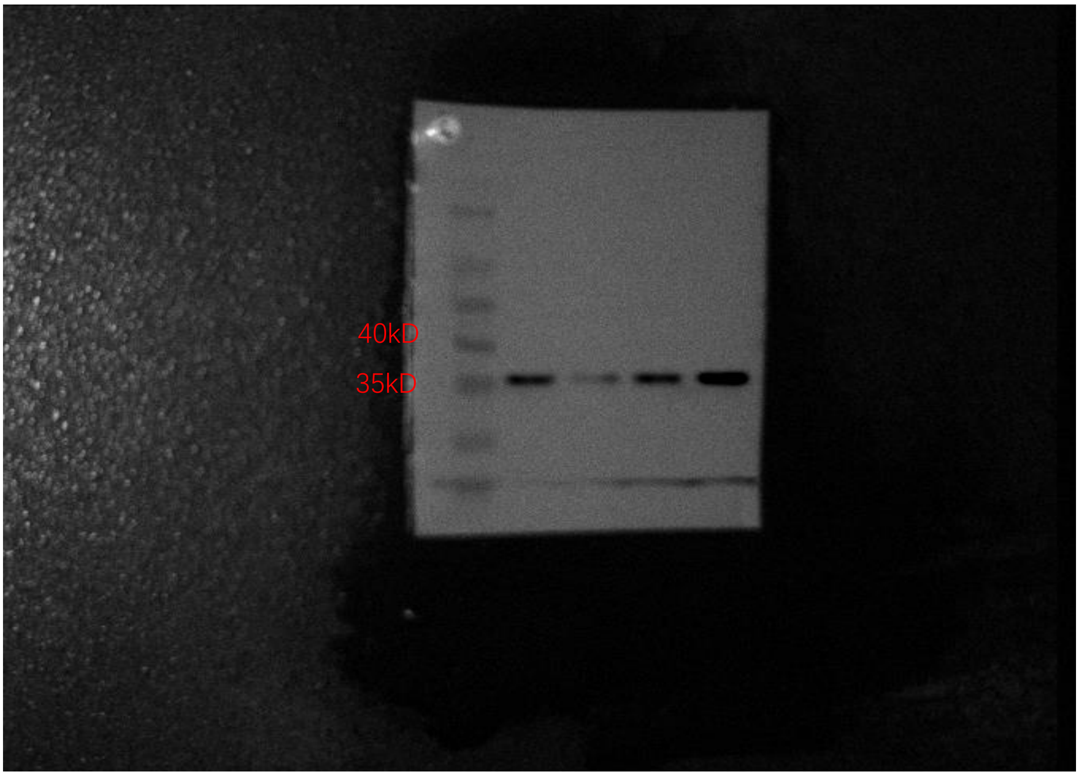

β-actin(42kD)

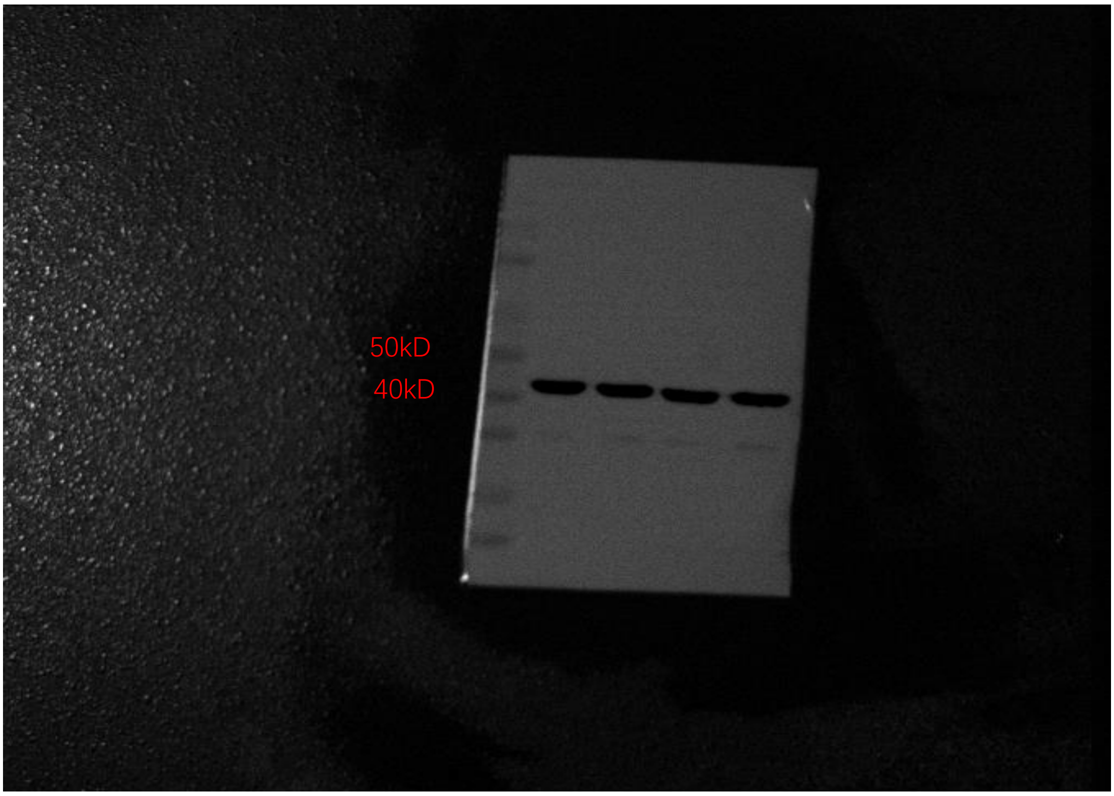

Figure 5A

MCF-7

ZWINT (35kD)

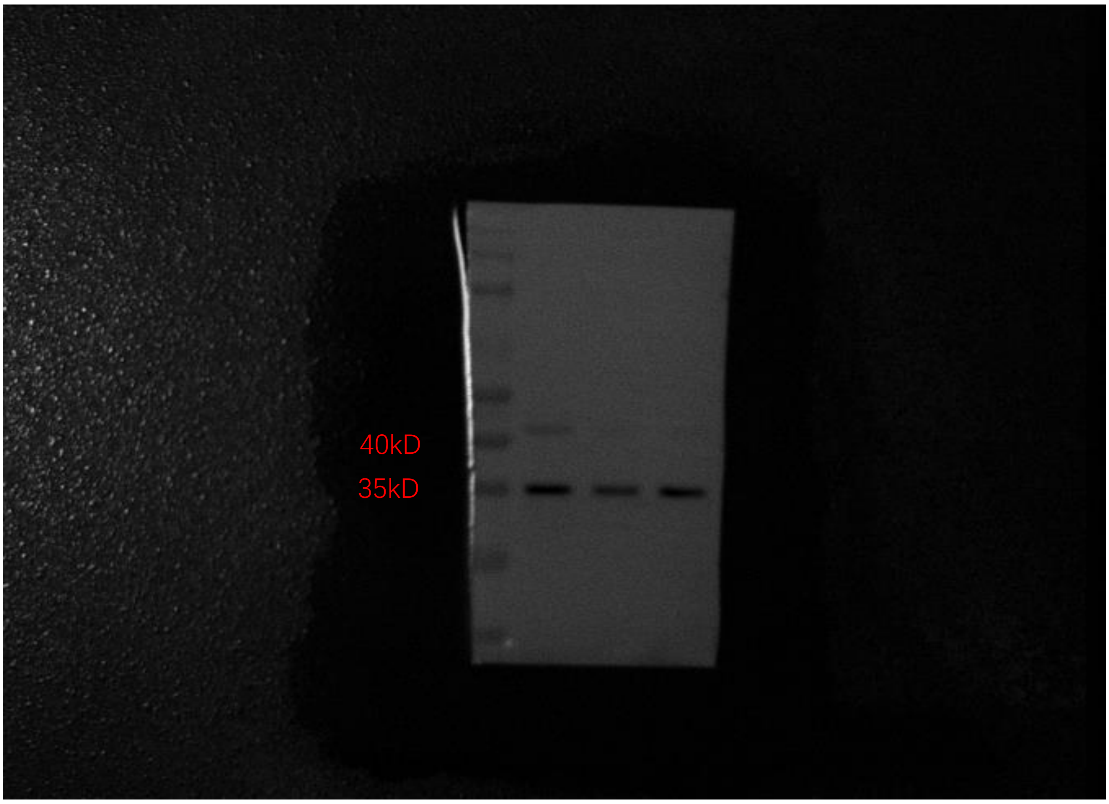

β-actin(42kD)

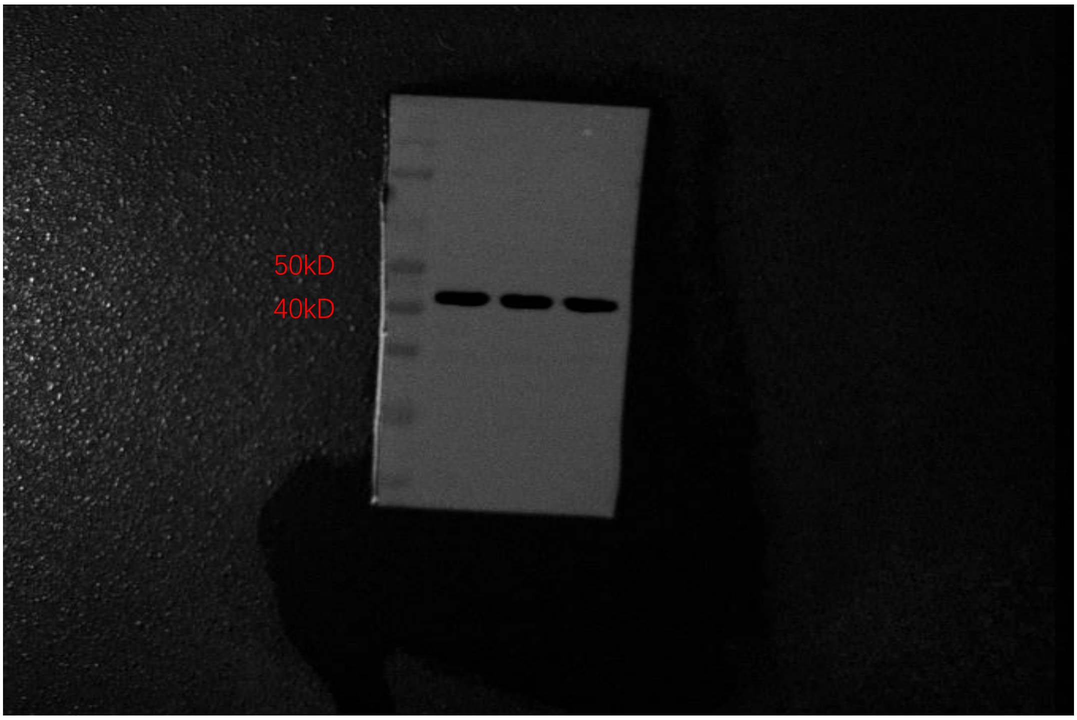

MDA-MB-231
ZWINT (35kD)

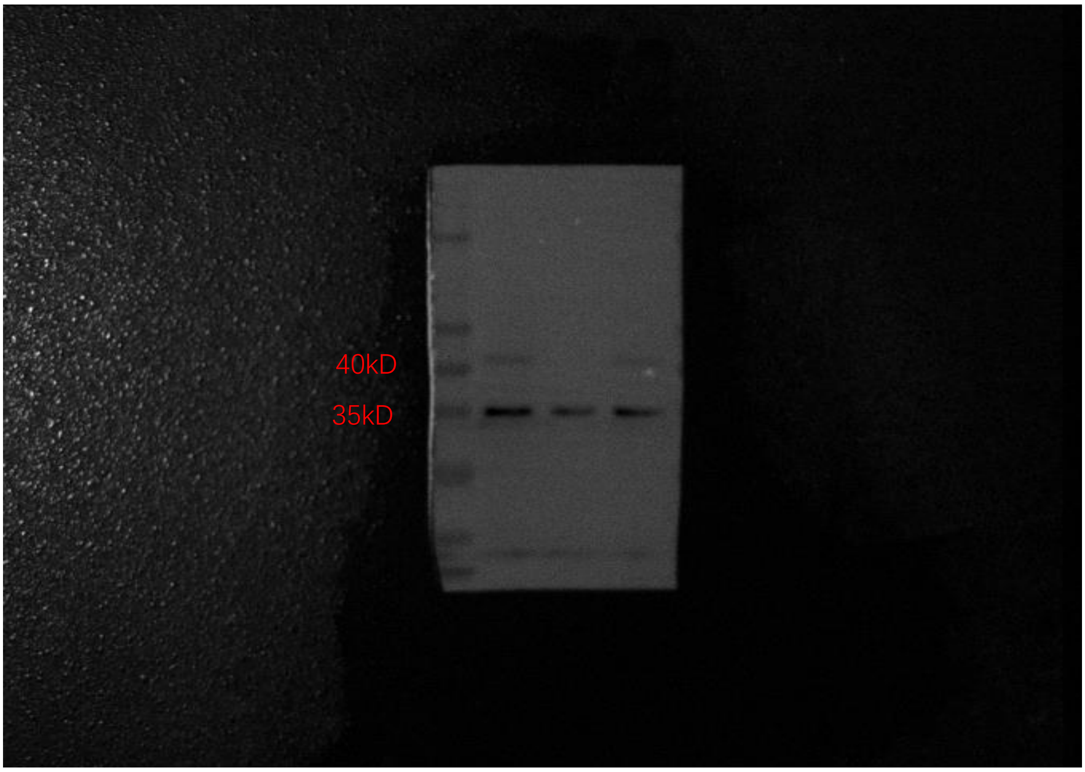

β-actin(42kD)

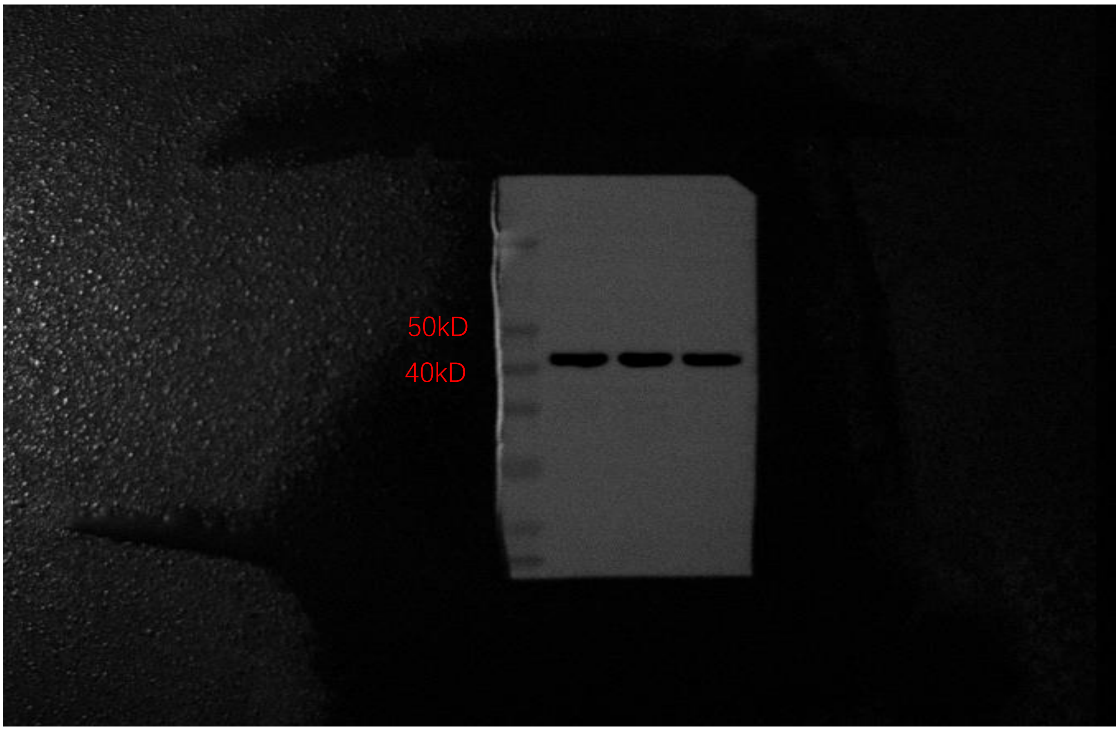

Figure 5F

MCF-7

p38(41kD)

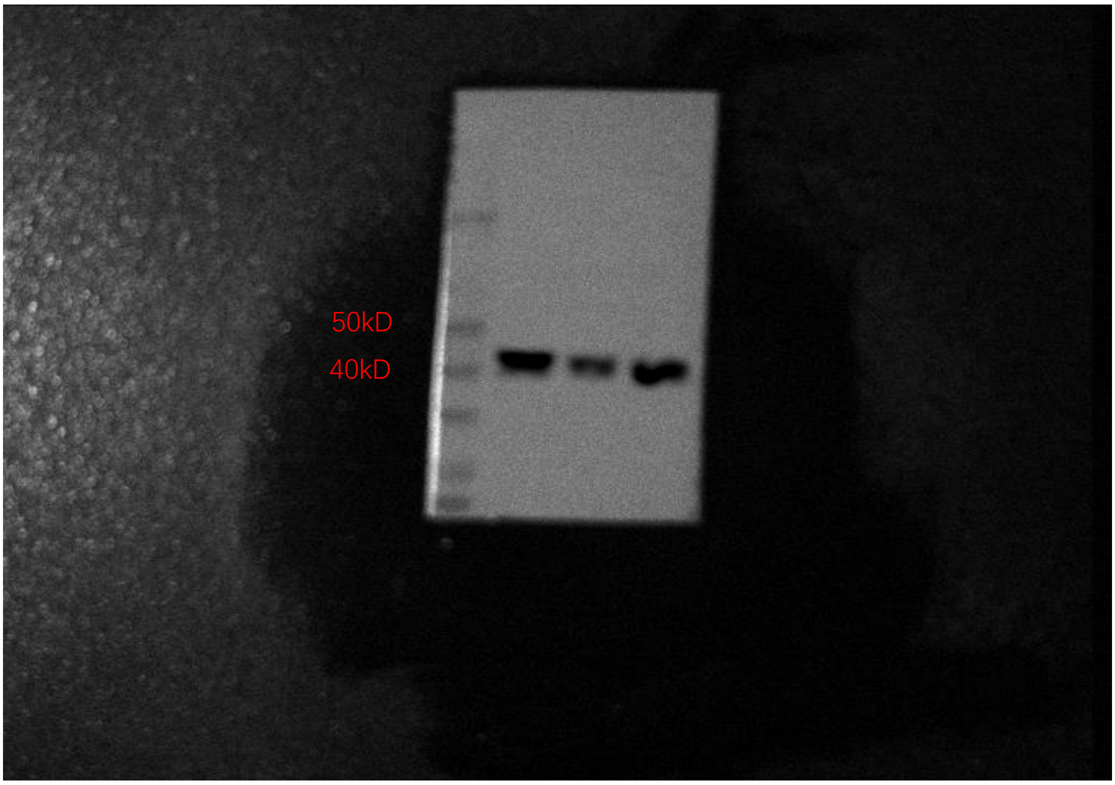

p-p38(41kD)

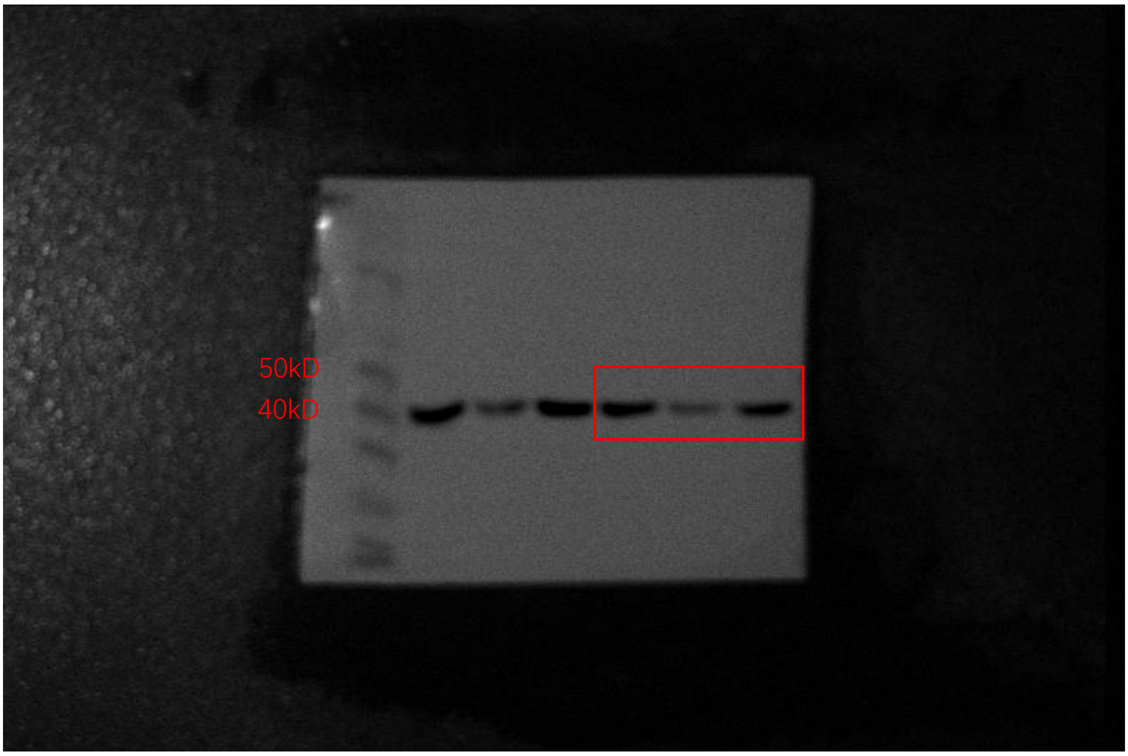

β-actin(42kD)

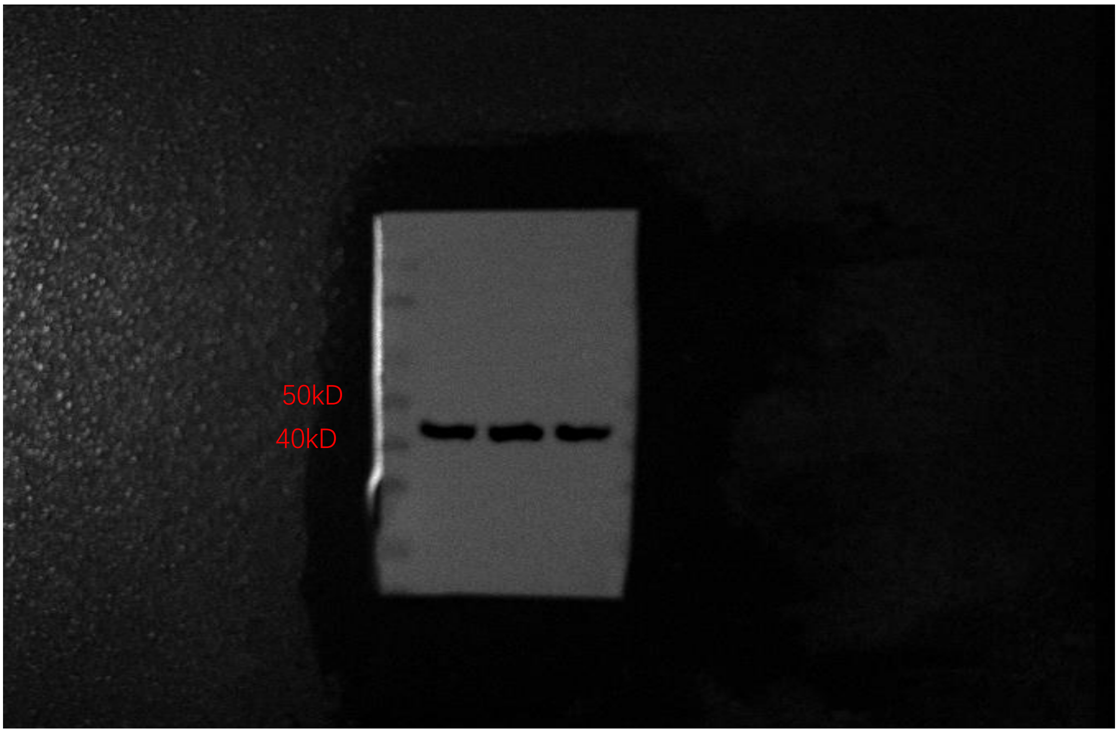

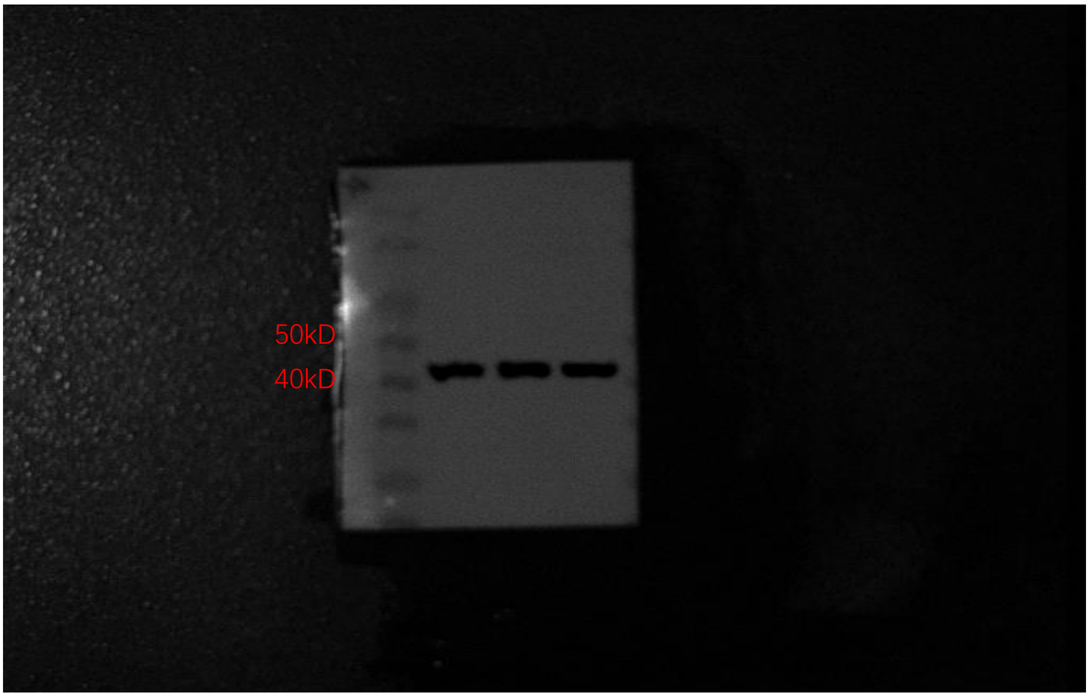

MDA-MB-231
p38(41kD)

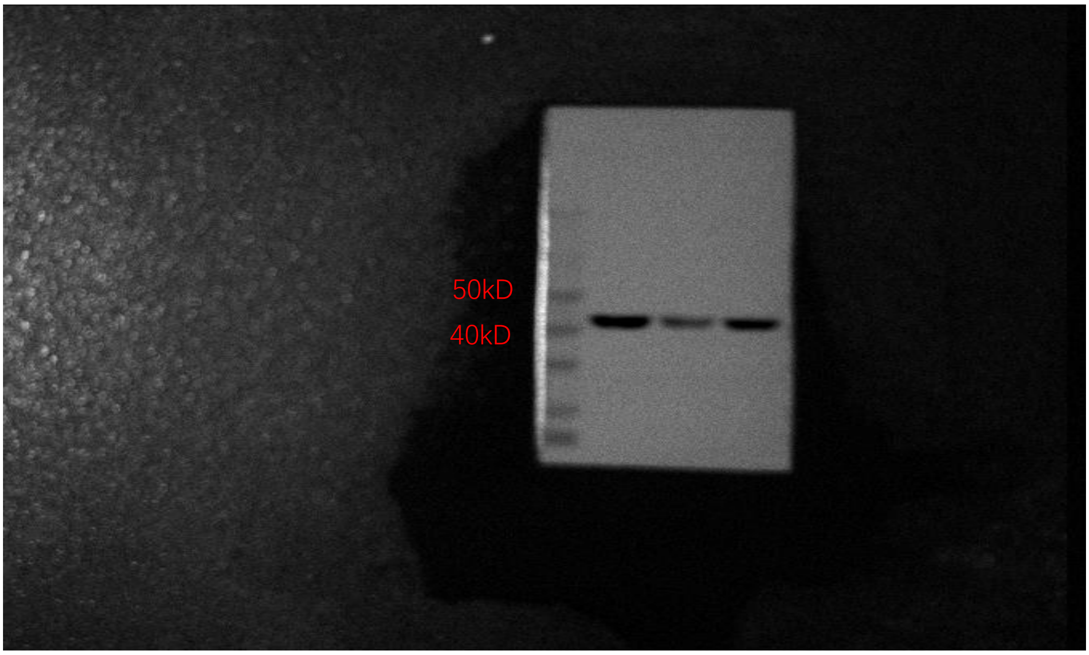

p-p38(41kD)

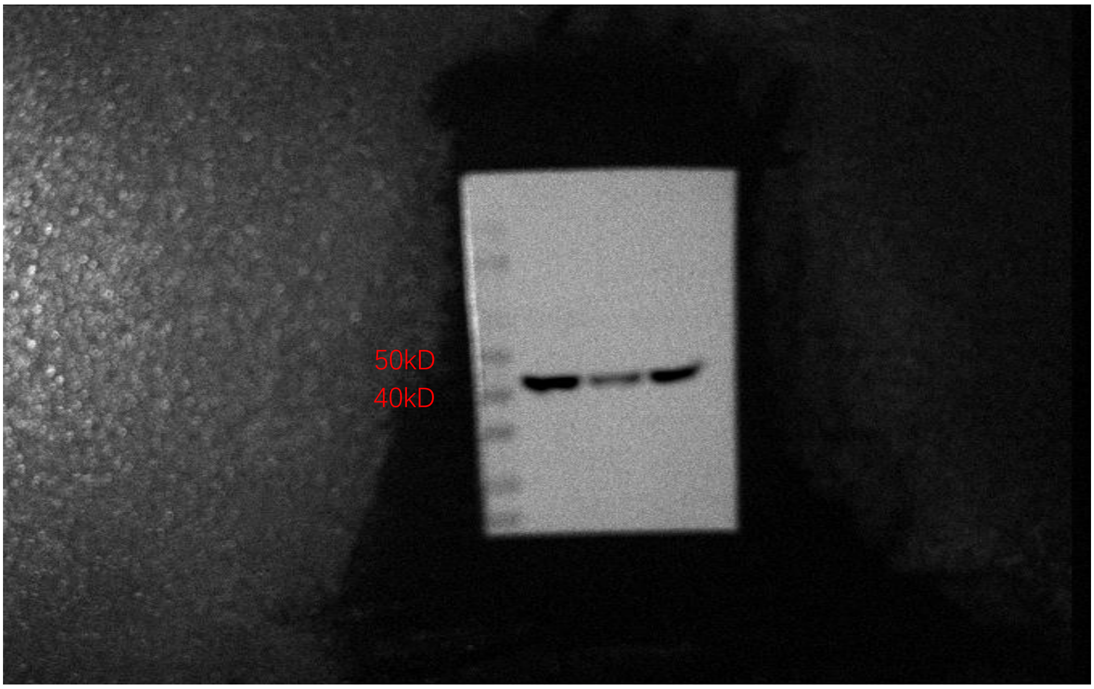

β-actin(42kD)

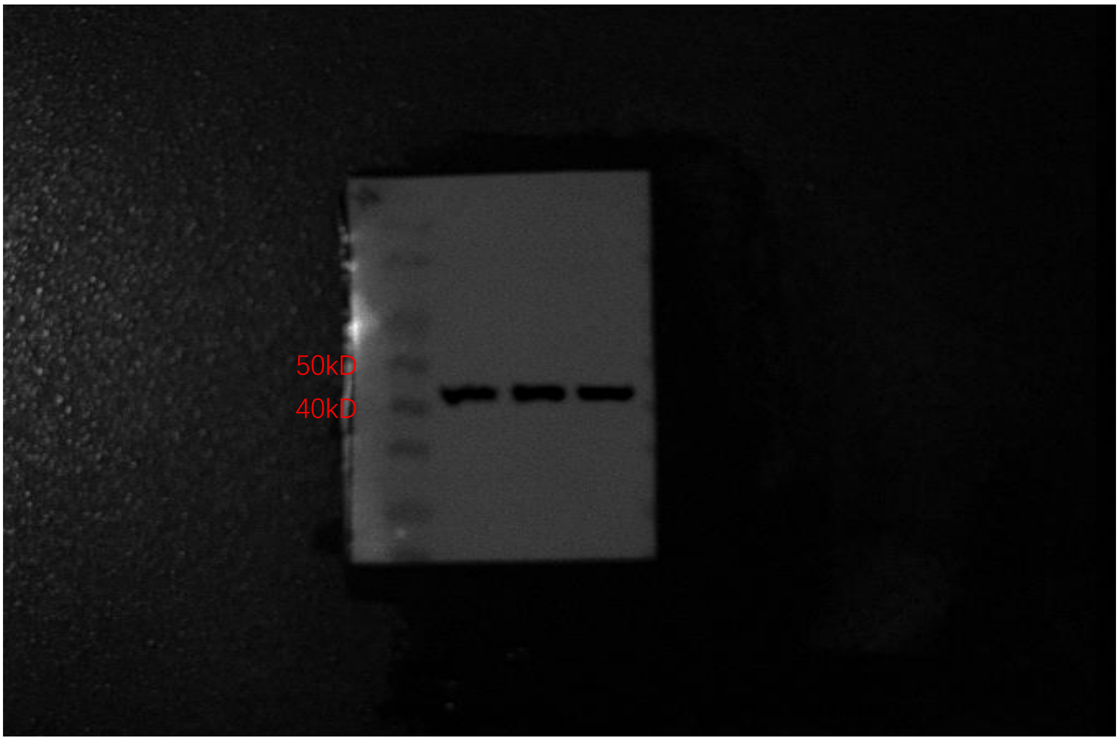

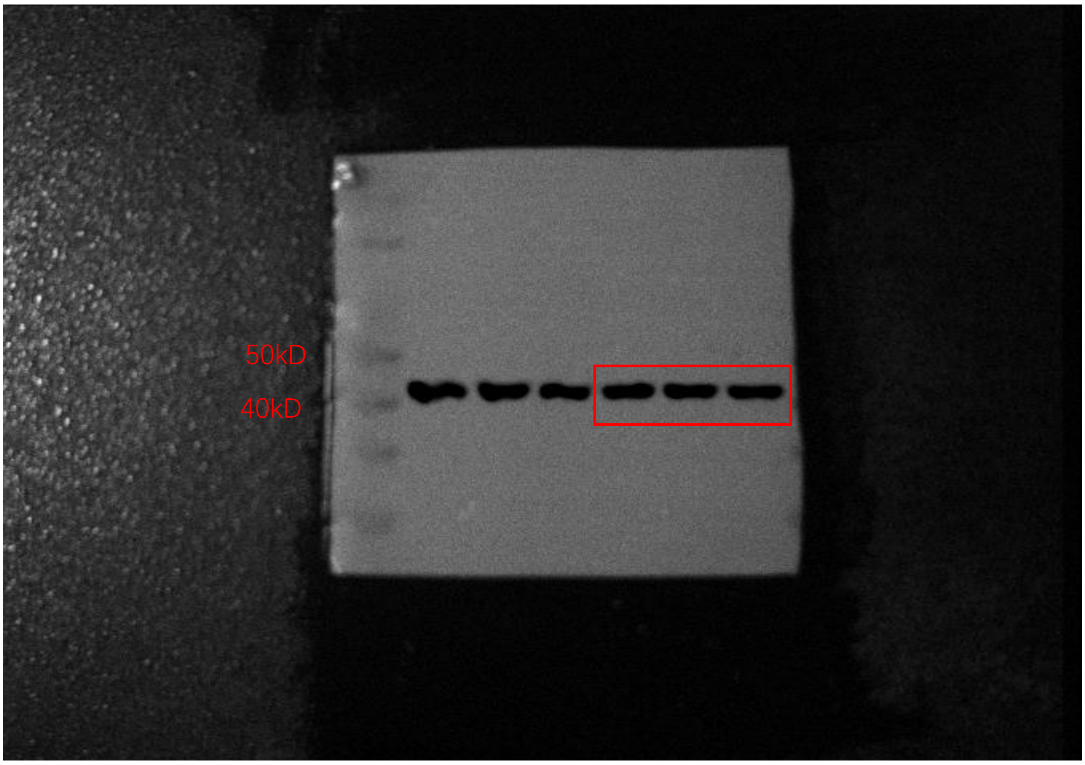

Supplementary figure 2

MCF-7

ZWINT (35kD)

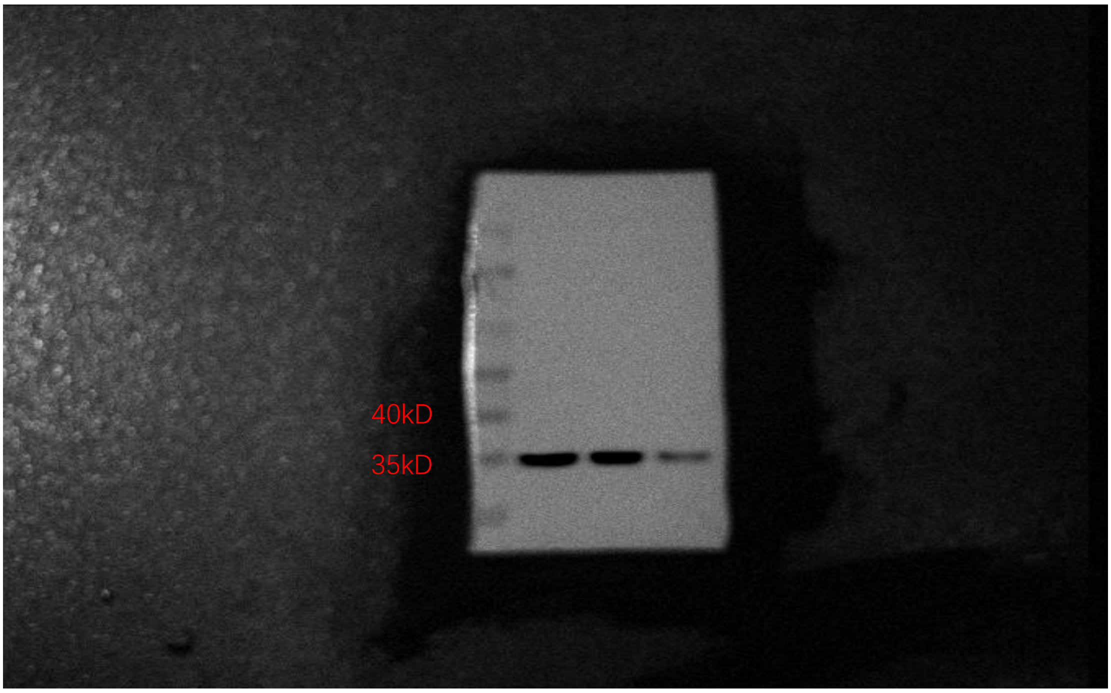

β-actin(42kD)

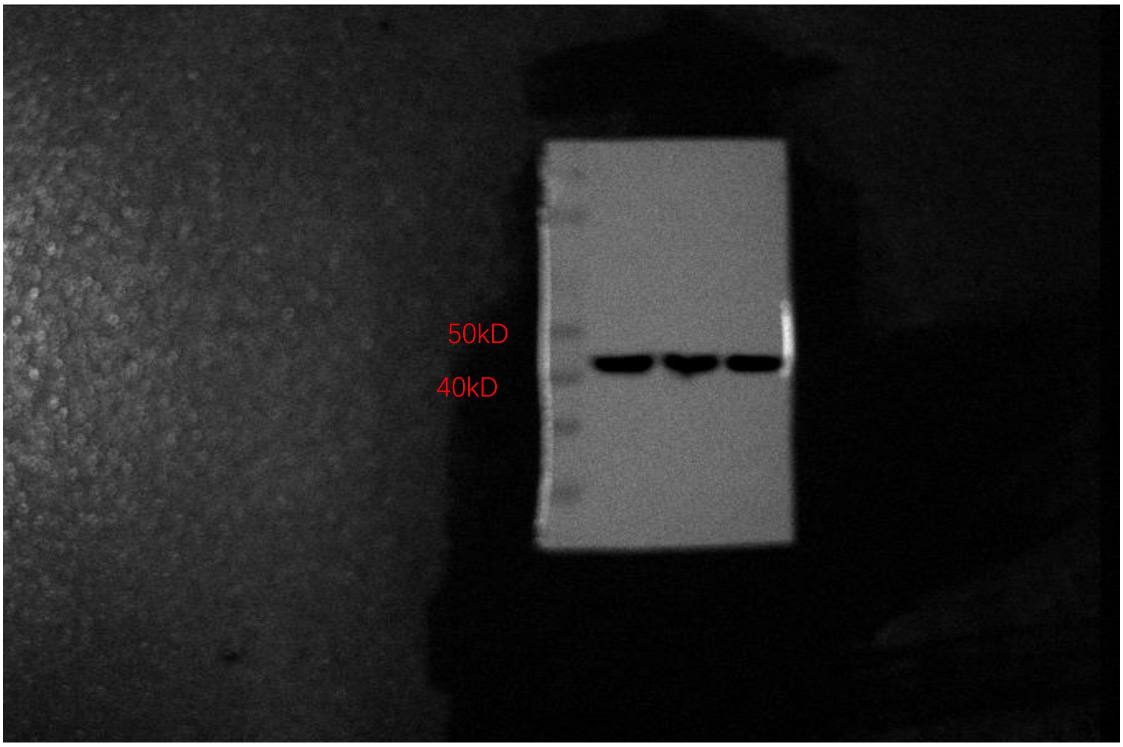

MDA-MB-231
ZWINT (35kD)

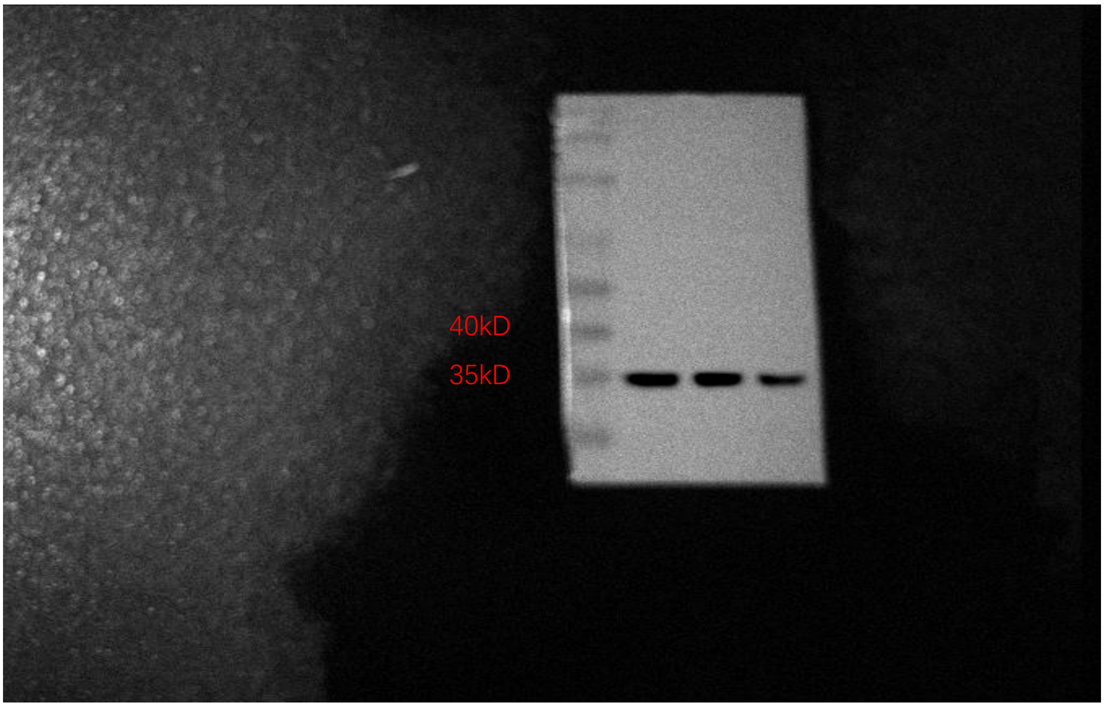

β-actin(42kD)

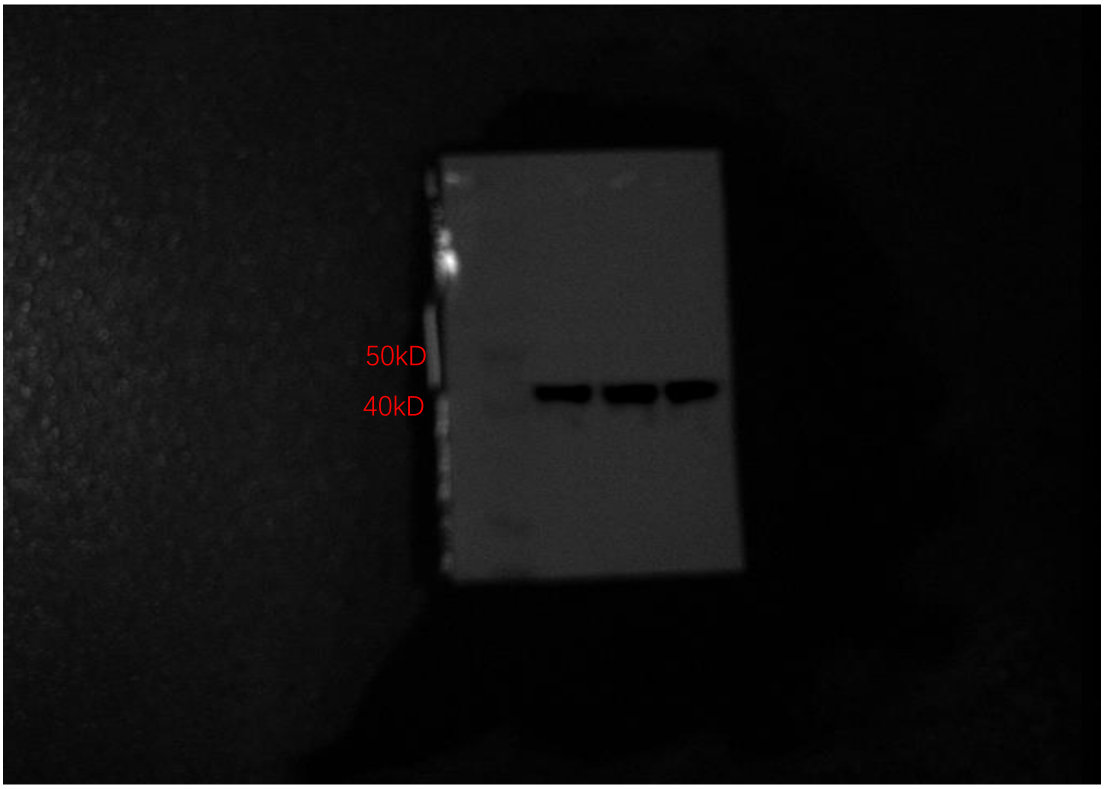
50kD
40kD
